# Mapping interpersonal-to-cognitive pathways with suicidal ideation: unforgiveness-based moderators within the interpersonal theory of suicide framework

**DOI:** 10.3389/fpsyg.2026.1749224

**Published:** 2026-04-09

**Authors:** Andy Po Sze Cheung, Sylvia Yuk Ching Lai Kwok, Minmin Gu, Yu Huang

**Affiliations:** 1 Department of Social and Behavioral Sciences, City University of Hong Kong, Kowloon, Hong Kong SAR, China; 2 Research Institute of Social Development, Southwestern University of Finance and Economics, Chengdu, China

**Keywords:** avoidance, Bayesian SEM, hopelessness, perceived burdensomeness, revenge, self-dysfunction, suicidal ideation, thwarted belongingness

## Abstract

**Background:**

Suicidal ideation among young adults is a significant public health concern. While the Interpersonal Theory of Suicide (ITS) provides a robust framework, the role of unforgiveness-based coping responses to interpersonal transgressions remains under-specified. Specifically, how strategies like avoidance, self-dysfunction, and revenge relate to the strength of ITS associations requires clarification. We examined cross-sectional associations among thwarted belongingness (TB), perceived burdensomeness (PB), hopelessness (HL), and suicidal ideation (SI), and whether these associations varied by unforgiveness-related coping (avoidance, self-dysfunction, revenge) within a moderated serial mediation framework.

**Methods:**

Standardized questionnaires were completed by 205 university-affiliated participants in Hong Kong. Given the cross-sectional design and zero-inflated outcome data, SI was modeled with Bayesian hurdle lognormal regression. Competing structural specifications were compared using WAIC/LOOIC to evaluate model plausibility.

**Results:**

Model comparisons favored a serial specification over a common-cause model. PB was robustly associated with both the presence and severity of SI. Moderator analyses indicated that avoidance and self-dysfunction were associated with a stronger link between TB and PB. Revenge motivation showed a complex pattern: it was associated with heightened hopelessness but reduced likelihood of reporting SI. In this non-clinical university sample, this pattern may reflect a form of emotional externalization, temporarily deflecting internalizing distress outward rather than conferring stable protection; this interpretation remains conditional on the cross-sectional design and sample characteristics.

**Discussion:**

Unforgiveness-based coping strategies appear to be associated with distinct points in the interpersonal-to-cognitive sequence. The “deflection” effect observed with revenge motivation should be interpreted with caution, likely representing an unstable externalization response rather than adaptive protection. Conversely, avoidance and self-dysfunction are linked to entrenched interpersonal distress. Integrating unforgiveness-based coping into suicide risk assessment offers a more granular understanding of risk. These findings highlight the value of assessing coping styles in youth-serving settings, suggesting that brief screening and low-intensity supports targeting specific coping mechanisms may be valuable for suicide prevention.

## 1 Introduction

Suicidal ideation is one of the most robust predictors of subsequent suicide attempts. Adolescent prevalence is nontrivial globally and locally: a meta-analysis of college samples estimated 22.3% lifetime suicidal ideation (Mortier et al., 2018), with a pooled lifetime prevalence in non-Western countries ≈18.7% (Lim et al., 2019). In Hong Kong, 21.8% of adolescents reported suicidal ideation (Chang et al., 2019), and rates are markedly higher (≈61%) among adolescents reporting non-suicidal self-injury (Cheung et al., 2013). The Interpersonal Theory of Suicide (ITS) (Van Orden et al., 2010) offers an influential framework for understanding suicidal desire. The sections that follow review the theoretical and empirical basis for these core constructs and related coping processes before specifying the models tested in the present study.

### 1.1 Thwarted belongingness and its link to perceived burdensomeness

The Interpersonal Theory of Suicide (ITS) posits that suicidal desire arises from TB and PB (Van Orden et al., 2010), but empirical findings for TB are mixed and generally weaker than for PB (Chu et al., 2017). One reason is that TB was originally framed as the “absence of meaningful connections,” which under-explains risk in high-density relational contexts (e.g., marriage, military; cf. Chu et al., 2017) where connections are present but function poorly. What may matter is not contact *per se* but whether ties meet the need to belong—feeling accepted, recognized, and valued (Baumeister and Leary, 1995). When existing ties are invalidating, critical, or hostile, they fail to meet this need and become sources of psychological pain (Rook, 2015), consistent with evidence linking negative social exchanges and insecure attachment to poorer mental health and suicide risk (Newsom et al., 2008; Sheftall et al., 2014).

Converging evidence suggests a cognitive bridge from unmet belonging (i.e., thwarted belongingness) to PB. Lower social connectedness is reliably associated with lower self-esteem (Lee and Robbins, 1998, 2000; see also Baumeister and Leary, 1995). Experimental work shows that negative performance feedback can elicit perceived burdensomeness and depress self-evaluations (Wirth et al., 2020). Over time, repeated “support failures” and rejection (Rook, 2015) may be encoded as negative core self-beliefs (“I am the problem”), in line with cognitive models (Beck, 1991; Wenzel and Beck, 2008), potentially consolidating PB as a more stable self-appraisal within the ITS architecture (Van Orden et al., 2010). Such beliefs are maintained by self-reinforcing interpersonal cycles characteristic of social anxiety and interpersonal dysfunction (Alden and Taylor, 2004; see also Davidson et al., 2011).

### 1.2 The mediating role of hopelessness

The ITS further proposes that suicidal desire is most likely when TB and PB are appraised as unchangeable, i.e., accompanied by hopelessness (Van Orden et al., 2010). A meta-analysis concluded that ITS tests have been “significantly hampered” by limited direct modeling of hopelessness as a key pathway component (Chu et al., 2017). Available evidence nonetheless supports its central role: classic and contemporary work links hopelessness to elevated suicidal ideation and behavior (Beck et al., 1974, 1999), and interpersonal stress combined with negative inferences confers risk for increased ideation (Joiner and Rudd, 1995). Within ITS samples, hopelessness interacts with or follows TB/PB in predicting ideation (Christensen et al., 2013; Kleiman et al., 2014), underscoring its theoretically central position as a cognitive appraisal that one’s interpersonal distress is intractable.

### 1.3 The proposed serial mediation model

On this basis, we specify a staged ITS-consistent sequence—TB → PB → Hopelessness → Suicidal Ideation—together with complementary routes in which TB contributes directly to hopelessness and, under acute social pain, may relate proximally to suicidal ideation. Despite being theoretically implied by ITS (Van Orden et al., 2010), this full sequence remains under-tested. Furthermore, given that cross-sectional associations may reflect shared underlying distress, we explicitly test this serial sequence against an alternative “common cause” model to evaluate its structural plausibility.

### 1.4 The moderating role of unforgiveness-based coping strategies

#### 1.4.1 The transgression-unforgiveness-coping pathway

We suggest that unforgiveness following an interpersonal transgression can create a sustained state of psychological distress that may alter how individuals appraise themselves and their relationships, thereby shaping ITS-consistent pathways to suicidal ideation. This process is theoretically grounded in several key concepts.

First, unforgiveness itself can be understood as a motivational state. Extending McCullough et al.’s (1997) model, which defines forgiveness as decreased motivation for avoidance and revenge, unforgiveness can be conceptualized as a state characterized by increased motivation for these responses. Wade and Worthington (2003) further operationalized unforgiveness as “the delayed emotions of resentment, hostility, hatred, bitterness, anger, and fear (in some combination) that arise after ruminating about a transgression” (p. 344), highlighting rumination as the mechanism that sustains and intensifies the unforgiveness state over time. Subsequent work has reinforced this conceptualization, positioning unforgiveness as a stress-related response with documented associations with poorer mental and physical health outcomes (Toussaint et al., 2015; Worthington et al., 2007), and temporal analyses indicate that transgression-related avoidance and revenge motivations follow dynamic trajectories that can either attenuate or consolidate depending on coping responses (McCullough et al., 2003). More recently, forgiveness has been identified as a moderator of the association between anger expression and suicidal behavior (Hirsch et al., 2012), and has been proposed as a positive psychotherapeutic mechanism for reducing suicide risk (Webb et al., 2015), reinforcing the clinical significance of the unforgiveness–coping pathway. Second, this state of unforgiveness is a primary source of psychache, or intolerable psychological pain (Shneidman, 1998). Shneidman argued that psychache arises from frustrated psychological needs, such as belonging and the avoidance of shame. Interpersonal transgressions directly threaten these needs, and the resulting unforgiveness may sustain this psychache, creating an urgent demand for a coping response.

Worthington and Scherer (2004) formalized this demand within a stress-and-coping framework, conceptualizing forgiveness as an emotion-focused coping strategy that reduces the stress of unforgiveness through reappraisal. When this coping process fails or is not engaged, the sustained unforgiveness state may be managed through alternative strategies—including the avoidance, self-dysfunction, and revenge responses examined in this study.

Building on this transgression–unforgiveness foundation, this study examines the moderating role of three specific coping strategies that represent distinct responses to this psychache: avoidance, self-dysfunction, and revenge.

#### 1.4.2 Moderators and their specific sequential mechanisms

Our theoretical framework extends Hirsch et al. (2017), who argue that the interpersonal constructs of the ITS—including both TB and PB—are fundamentally intrapersonal in nature. Although PB is socially framed as a belief that one is a liability to others, Hirsch et al. (2017) posit that it is driven by “a plethora of self-impugning attributions (i.e., negative self-evaluations in which individuals blame themselves for interpersonal failures and view themselves as fundamentally flawed),” including self-liability and self-hate. Consistent with the psychache framework introduced above (section 1.4.1), burdensomeness can thus be understood as an active manifestation of unbearable psychological pain rooted in shame, guilt, and self-loathing.

This perspective aligns with the bio-cognitive-interpersonal hypothesis of Shahar et al. (2020), who propose that self-criticism acts as a persistent internal stressor that—via ruminative brooding and interpersonal stress generation—erodes interpersonal security and fuels suicidal urges. O’Neill et al. (2021) provide empirical support for this pathway, demonstrating that maladaptive self-criticism robustly predicts suicide probability.

The current study examines how specific unforgiveness-based coping strategies may differentially engage these self-impugning processes at distinct nodes of the ITS pathway. Self-dysfunction may directly amplify identity-based shame that consolidates burdensomeness beliefs; avoidance may maintain self-critical schemas by precluding corrective social experience; and revenge motivation may redirect self-directed distress outward, altering downstream cognitive links. These pathway-specific mechanisms are elaborated in the sections that follow.

#### 1.4.3 Avoidance as moderator: the withdrawal pathway

When unforgiveness manifests through avoidance, individuals may cope with transgression-related pain by withdrawing from social connections. However, research suggests this strategy may paradoxically intensify the very problems it seeks to solve. Specifically, avoidance may strengthen the avoidant motivation component identified by McCullough et al. (1997), potentially contributing to a self-perpetuating cycle where social withdrawal increases isolation, which may in turn amplify feelings of defeat and thwarted belongingness.

Alden and Taylor (2004) suggest that in self-perpetuating interpersonal cycles, individuals with high social anxiety develop negative schemas leading them to avoid social interaction while anticipating rejection from others. This negative feeling and psychological pain may further enhance dysfunctional interpersonal styles, which function as distorted protection against rejection but may paradoxically increase rejection probability and vulnerability to social phobia development (Alden and Taylor, 2004). We propose that the moderators and their influence on suicide pathways may operate through multiple interconnected steps: First, individuals experiencing transgression-related unforgiveness may choose avoidance as a coping strategy to protect themselves from further harm. However, this avoidance behavior may lead to increased social isolation, as Davidson et al. (2011) found that interpersonal dysfunction correlates with suicidal ideation. Subsequently, this isolation may be linked to heightened defeat feelings, which Tarsafi et al. (2015) define as “subjective feelings of being brought down in status or fortune” (p. 777). These defeat feelings may then amplify perceived burdensomeness as individuals begin to internalize their social isolation as evidence of their worthlessness to others. Finally, this amplified perceived burdensomeness may create a stronger association with hopelessness about one’s interpersonal status, potentially resulting in elevated suicidal ideation.

This pathway may be particularly destructive because avoidance may prevent the natural healing that could occur through positive social interactions, thereby potentially maintaining the unforgiveness state indefinitely. Williams et al. (2006) note that avoidance functions as an emotional regulation mechanism to prevent the retrieval of negative memories, particularly traumatic ones, but this protective function may paradoxically maintain the psychological conditions that foster suicidal ideation.

#### 1.4.4 Self-dysfunction as moderator: the internal conflict pathway

Self-dysfunction may represent a more complex moderating mechanism because it may involve the simultaneous pursuit of belonging needs and self-protective strategies. According to the DSM-5 AMPD criteria, self-dysfunction encompasses both identity impairment and self-direction deficits (Gamache et al., 2019). When unforgiveness combines with self-dysfunction, individuals may experience internal contradictions between their need to belong and their maladaptive self-protective behaviors.

Miller (1999) suggests that dysfunctional relationships relate to personal dysfunction, which consists of insecure attachment, jealousy, loneliness, or depression—variables that correlate with thwarted belongingness and perceived burdensomeness. Miller further explained that troubled relationships can emerge from interactive contributions of individuals without apparent personal dysfunctions, influenced by betrayals, problematic attributions, and poor nonverbal communication.

We hypothesize that these processes may unfold as follows: unforgiveness may heighten identity confusion and impair self-direction as individuals struggle to reconcile their desire for connection with protective withdrawal tendencies. This internal conflict may then manifest as contradictory behaviors where individuals simultaneously seek connection while engaging in self-sabotaging behaviors that push others away. These contradictory behaviors may subsequently increase interpersonal dysfunction, as Davidson et al. (2011) found that interpersonal dysfunction is prevalent within close relationships of socially anxious individuals. This interpersonal dysfunction may then amplify perceived burdensomeness specifically through identity-related shame, where individuals begin to view themselves as fundamentally flawed and burdensome to others. The identity-based shame may heighten hopelessness about one’s capacity for meaningful relationships, potentially contributing to suicidal ideation.

Crucially, self-dysfunction may moderate this relationship by creating identity-based shame that could specifically amplify the “I am a burden” cognitions central to perceived burdensomeness. This may be significant because the shame is not merely about specific behaviors but about one’s fundamental identity and worth as a person.

#### 1.4.5 Revenge as moderator: the retaliatory control pathway

Revenge motivation may represent the most socially engaged yet destructive coping response to unforgiveness. Building on A. Adler’s (1979) framework, revenge-motivated individuals may view suicide as a means to ‘hurt others by dreaming himself into injuries or by administering them to himself’ (p. 252). Adler (1980) expanded this concept, describing revenge as passive-aggressive behavior representing an “illusory victory” likely practiced extensively in childhood. This pathway may be unique because it transforms perceived burdensomeness from a source of shame into a weapon of retaliation.

We propose that the moderators and their influence on suicide pathways may operate through a distinctive transformation process: First, unforgiveness may be characterized by sustained anger and resentment toward those perceived as responsible for the transgression. This sustained anger may manifest as active revenge motivation, where individuals begin to fantasize about ways to retaliate against those who have hurt them. Subsequently, this revenge motivation may facilitate a cognitive reframing of suicide as retaliation, where death becomes conceptualized not as escape but as punishment for others. This reframing may be associated with a distorted amplification of perceived burdensomeness, where individuals begin to think “my death will punish them” or “they will suffer when I’m gone.” This distorted perception of burdensomeness may then co-occur with hopelessness about interpersonal relationships, but with a vengeful twist where the hopelessness is accompanied by anticipation of posthumous satisfaction. Finally, this complex emotional state may be expressed as suicidal ideation that is motivated by both escape and retaliation.

From this theoretical perspective, revenge may moderate the PB–suicidal ideation relationship by transforming burdensomeness from a purely negative self-perception into a perceived tool for interpersonal control. However, rather than acting as a healthy buffer, we propose that revenge motivation may function as a mechanism of “externalization” or “deflection”—temporarily directing distress outward and masking the immediate link to suicidal ideation, potentially at the cost of long-term interpersonal functioning. Research indicates some individuals fear dying alone and desire connection with others (Ozawa-de Silva, 2008), and the calculation of potential suicide benefits may resonate with dysfunctional attitudes involving seeking approval, pleasing others, or avoiding appearing weak.

### 1.5 Theoretical framework and hypothesized models

**FIGURE 1:**
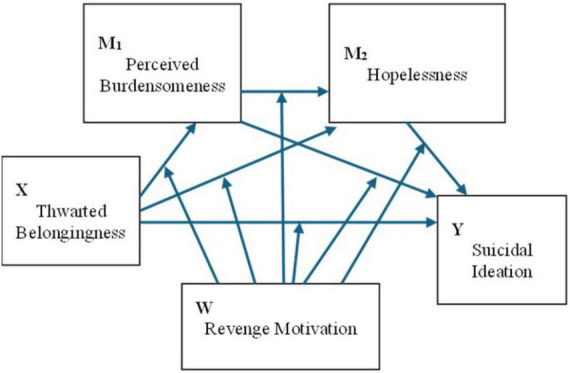
Conceptual diagram of Model 1 (single-moderator serial mediation). Revenge motivation (W) is specified as the sole moderator across multiple links in the serial pathway from thwarted belongingness (X) through perceived burdensomeness (M_1_) and hopelessness (M_2_) to suicidal ideation (Y). Arrows represent hypothesized directional associations.

Despite the robust evidence base for the ITS framework, less is known about how cognitive and motivational responses to interpersonal harm—particularly unforgiveness-based coping—may shape the strength of ITS-consistent associations. To address this gap, the present study integrates unforgiveness-based coping responses as node-specific moderators within an ITS-consistent pathway model. Grounded in the Interpersonal Theory of Suicide (ITS) (Van Orden et al., 2010), we conceptualize a staged, cognitive–affective sequence from interpersonal strain to suicidal ideation. When people feel that they do not belong (thwarted belongingness), they may be more likely to appraise themselves as a burden to others (perceived burdensomeness). These self-appraisals are hypothesized to be associated with greater hopelessness about change, which, in turn, is expected to relate to suicidal ideation. Consistent with ITS, we also retain a complementary route in which thwarted belongingness contributes directly to hopelessness (e.g., via exclusion and self-devaluation appraisals) and a proximal route from thwarted belongingness to suicidal ideation (e.g., under acute social pain; see Figure 1).

Building on the transgression–unforgiveness literature, we position coping responses as node-specific moderators of this sequence (Enright and Fitzgibbons, 2000; Hirsch et al., 2017; McCullough and Hoyt, 2002; McCullough et al., 1997, 1998; Shneidman, 1996, 1998; Spielberger, 1999; Tangney et al., 1996). Avoidance motivation and self-dysfunction are theorized to intensify the translation from thwarted belongingness to perceived burdensomeness, through mechanisms such as withdrawal, identity-based shame, and self-critical schemas that consolidate “I am a burden” beliefs. Revenge motivation is theorized to shape downstream links by channeling distress outward rather than inward: it may amplify the impact of thwarted belongingness on hopelessness while attenuating internalization-prone links, consistent with distinctions between internalization and externalization of psychache (Shneidman, 1996, 1998; Spielberger, 1999; Tangney et al., 1996).

**FIGURE 2:**
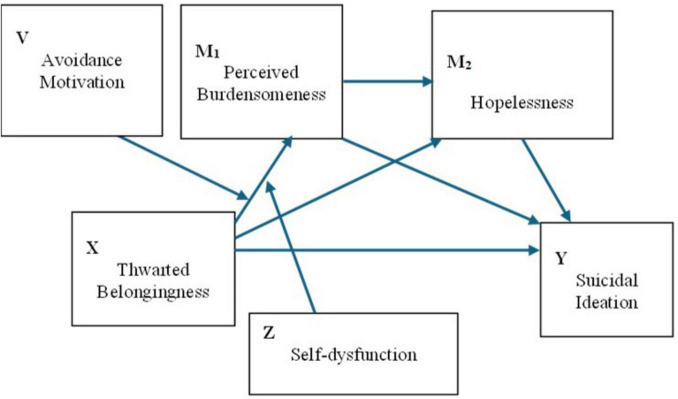
Conceptual diagram of Model 2 (dual first-stage moderation). Avoidance motivation (V) and self-dysfunction (Z) jointly moderate the initial link from thwarted belongingness (X) to perceived burdensomeness (M_1_). Downstream pathways through hopelessness (M_2_) to suicidal ideation (Y) are specified without moderation.

**FIGURE 3:**
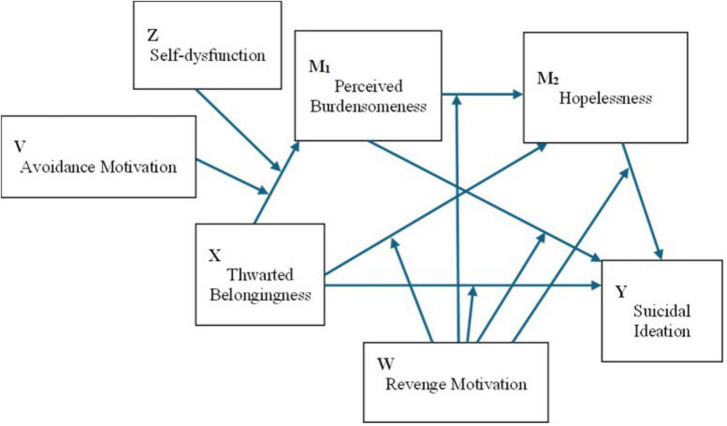
Conceptual diagram of Model 3 (integrated multi-node moderation). Avoidance motivation (V) and self-dysfunction (Z) moderate the first-stage link from thwarted belongingness (X) to perceived burdensomeness (M_1_). Revenge motivation (W) moderates downstream links, including paths to hopelessness (M_2_) and the direct path to suicidal ideation (Y).

To summarize, the present study addresses three interrelated aims. First, we test and ITS-consistent serial mediation sequence (TB → PB → HL → SI) and compare it against a common-cause alternative to evaluate structural plausibility. Second, we examine whether unforgiveness-based coping strategies—avoidance, self-dysfunction, and revenge motivation—moderate specific nodes of this sequence, with avoidance and self-dysfunction hypothesized to strengthen the early TB → PB translation and revenge motivation hypothesized to shape downstream links. Third, we employ a Bayesian hurdle modeling approach to separately examine factors associated with the presence versus the severity of suicidal ideation. Three competing model specifications (Figures 1–3) are described below and evaluated in the Results.

#### 1.5.1 Hypothesized models

Model 1 (single-moderator serial model). Revenge motivation is specified as the sole moderator across multiple links (Figure 1). In this model, we test whether revenge motivation alters: (a) the translation from thwarted belongingness to perceived burdensomeness, (b) the complementary path from thwarted belongingness to hopelessness, and (c) the influence of each mediator on suicidal ideation (i.e., perceived burdensomeness to suicidal ideation and hopelessness to suicidal ideation). The unmoderated backbone remains the sequential path from thwarted belongingness through perceived burdensomeness and hopelessness to suicidal ideation, with complementary routes from thwarted belongingness to hopelessness and to suicidal ideation.

Model 2 (dual first-stage moderation). Avoidance motivation and self-dysfunction jointly moderate the initial link from thwarted belongingness to perceived burdensomeness (Figure 2), i.e., withdrawal- and identity-based self-processing are expected to strengthen this translation. Downstream pathways through hopelessness and to suicidal ideation are specified without moderation in this model.

Model 3 (integrated multi-node moderation). Avoidance motivation and self-dysfunction again moderate the link (Figure 3) from thwarted belongingness to perceived burdensomeness. Revenge motivation is positioned to moderate downstream links—specifically, the path from thwarted belongingness to hopelessness, the path from perceived burdensomeness to hopelessness, and the direct path from thwarted belongingness to suicidal ideation—reflecting theoretical distinctions between outward versus inward directions of distress. These model specifications constitute hypotheses to be tested; model estimation and comparisons are reported in the Results.

## 2 Materials and methods

This study utilized a cross-sectional survey design. Data were collected from 205 university-affiliated participants (57 males, 148 females; Mean age = 21.73, SD = 4.14; age range: 17–39) in Hong Kong. Recruitment employed convenience and snowball sampling methods via university channels and social networks. Ethical approval was granted by the Research Ethics Review Committee of the affiliated university. All participants provided informed consent via a secure online platform prior to participation. As participants were drawn from a non-clinical, university-affiliated population, the resulting sample is best characterized as a community convenience sample; generalization to clinical populations or individuals with acute suicidal risk should be made with caution.

Demographic characteristics are detailed in Table 1. The sample was predominantly female (72.2%) and non-religious (73.2%). The majority had attained a college education (79.5%). Regarding family background, 74.1% of participants’ parents were married, and family income distributions varied, with 25.4% of participants unsure of their specific household income bracket.

**TABLE 1:** Demographic variable of participant (*N* = 205).

Variable	Category	Frequency (%)	Mean (SD)
Gender	Male	57 (27.8)	
	Female	148 (72.2)	
Age			21.73 (4.14)
Education	Primary	8 (3.9)	
	Secondary	10 (4.9)	
	College	163 (79.5)	
	Undergraduate	24 (11.7)	
	Master or above	0 (0%)	
	Other	0 (0%)	
Religion	None	150 (73.2)	
	Catholic	5 (2.4)	
	Christian	36 (17.6)	
	Buddhism	7 (3.4)	
	Taoism	4 (2.0)	
	Other	3 (1.5)	
Parent marital status	Married	152 (74.1)	
	Remarriage	5 (2.4)	
	Divorce	26 (12.7)	
	Separation	4 (2.0)	
	Cohabitation	6 (2.9)	
	Other	12 (5.9)	
Family income	Below $10,000	5 (2.4)	
	$10,001–$30,000	60 (29.3)	
	$50,001 or above	44 (21.5)	
	Do not know	52 (25.4)	
		**Father frequency (%)**	**Mother frequency (%)**
Education	No formal education	3 (1.5)	4 (2.0)
	Primary	39 (19)	29 (14.1)
	Secondary 1–3	37 (18)	49 (23.9)
	Secondary 4–5	28 (13.7)	32 (15.6)
	Secondary 6–7	25 (12.2)	26 (12.7)
	Technical school or vocational training	12 (5.9)	10 (4.9)
	College or above	56 (27.3)	51 (24.9)
	Other	5 (2.4)	4 (2.0)

### 2.1 Measuring instruments

The Chinese version of the Interpersonal Needs Questionnaire (INQ) (Van Orden et al., 2012) was used to assess thwarted belongingness and perceived burdensomeness, two key constructs related to suicidal desire. The scale consists of 15 items rated on a 7-point Likert scale (1 = not at all true for me, 7 = very true for me), with higher scores reflecting greater levels of the relevant constructs. Example items include “These days, I feel disconnected from other people” for thwarted belongingness, and “These days the people in my life would be better off if I were gone” for perceived burdensomeness. The Chinese version was translated and back-translated by bilingual researchers. In this study, Cronbach’s alpha was 0.92 for the full scale, 0.93 for perceived burdensomeness, and 0.89 for thwarted belongingness.

Self-dysfunction was measured using the Chinese version of the Self and Interpersonal Functioning Scale (SIFS) (Gamache et al., 2019), which adopts a 5-point Likert scale (0 = does not describe me at all, 4 = describes me totally). Higher scores reflect greater self-dysfunction. Example items include “My self-esteem is easily affected if I experience failures or disappointments” and “Sometimes I don’t understand why I behaved in a certain way or why I made some decisions.” The self-dysfunction subscale demonstrated acceptable reliability in the present study (Cronbach’s alpha = 0.72).

Hopelessness was measured using the Chinese version of the 10-item Beck Hopelessness Scale (BHS; Beck et al., 1974), which uses a 4-point Likert scale (1 = very false, 4 = very true). Higher scores indicate greater hopelessness. An example item is “I look forward to the future with hope and enthusiasm.” In this study, the scale demonstrated excellent reliability (Cronbach’s alpha = 0.92).

Forgiveness-related motivations were assessed with the Chinese version of the Transgression-Related Interpersonal Motivations Inventory (TRIM; McCullough et al., 1998), which includes two subscales: avoidance motivation and revenge motivation. The 12 items are rated on a 5-point Likert scale (1 = strongly disagree, 5 = strongly agree), with higher scores indicating stronger avoidance or revenge motivation. Example items include “I withdraw from him/her” for avoidance motivation and “I’ll make him/her pay” for revenge motivation. The scale exhibited good internal consistency in the current study, with Cronbach’s alpha values of 0.87 for the full scale, 0.88 for avoidance motivation, and 0.92 for revenge motivation.

Suicidal ideation was assessed using the adapted Chinese version (Sun et al., 2006) of the first five items from the Scale for Suicidal Ideation (SSI; Beck et al., 1979). These five items screened for the presence and severity of suicidal thoughts, including aspects such as “wish to live,” “wish to die,” “desire to make an active suicide attempt,” “reasons for living/dying,” and “passive suicidal desire.” Items relating to wish to live, wish to die, and active suicide desire were rated on a 4-point scale (0–3), while items on reasons for living/dying and passive suicidal desire used a 3-point scale (0–2). Higher total scores indicated more severe suicidal ideation. In the present study, the adapted scale showed good reliability (Cronbach’s alpha = 0.84).

### 2.2 Data analysis plan

To evaluate an Interpersonal Theory of Suicide (ITS)–consistent interpersonal-to-cognitive sequential mechanism while also accommodating the zero-inflated, right-skewed distribution of suicidal ideation (SI) in a non-clinical sample, we implemented a two-stage Bayesian analytic strategy.

- Stage 1 (Structural model testing): Bayesian structural equation modeling (SEM) was used to test the hypothesized serial pathway and its node-specific moderation patterns within the ITS framework (Hayes, 2017; Merkle and Rosseel, 2018; Van Orden et al., 2010).
- Stage 2 (Distribution-appropriate outcome modeling): A Bayesian hurdle lognormal model was used to estimate associations with SI in a way that explicitly models (a) the probability of any ideation versus none and (b) the intensity of ideation among those reporting non-zero SI (Bürkner, 2017; Hu et al., 2011).

All analyses were conducted in R. SEMs were estimated using blavaan (Merkle and Rosseel, 2018), and hurdle models were estimated using brms (Bürkner, 2017). Convergence was assessed using rank-normalized R-hat and effective sample size (ESS), using conventional thresholds (R-hat ≤ 1.01; ESS > 400 as a minimum benchmark) (Vehtari et al., 2021).

#### 2.2.1 Stage 1: structural model testing (Bayesian SEM)

To enhance transparency and align with Hayes’ conditional process approach, we estimated three moderated mediation structural equation models (SEMs) that map onto widely used PROCESS templates (Hayes, 2017). Across all models, we specified the same ITS-consistent backbone: a serial mediation pathway (TB → PB → HL → SI), together with complementary direct paths (TB → HL and TB → SI). Using PROCESS-style notation, variables were defined as X = thwarted belongingness (TB), M1 = perceived burdensomeness (PB), M2 = hopelessness (HL), and Y = suicidal ideation (SI). The moderators were W = revenge motivation (RM), V = avoidance motivation (AM), and Z = self-dysfunction (SF) (Hayes, 2017).

We compared three competing moderation specifications:

1. Model 1 (PROCESS Model 92; single-moderator moderated serial mediation). Revenge motivation (W) was specified as the sole moderator operating across multiple links in the moderated serial mediation system (e.g., moderation of X→M paths, M→Y paths, and/or the direct X→Y path), while retaining the serial backbone (TB → PB → HL → SI) and complementary paths (TB → HL; TB → SI) (Hayes, 2017).
2. Model 2 (dual first-stage moderation; PROCESS Model 9–style). Avoidance motivation (V) and self-dysfunction (Z) were specified as first-stage moderators on the X→M1 path (TB → PB), whereas downstream links (PB → HL, PB → SI, HL → SI) were unmoderated (Hayes, 2017).
3. Model 3 (integrated moderated serial mediation; final model). Avoidance motivation (V) and self-dysfunction (Z) jointly moderated the first-stage translation from TB to PB (X→M1). Revenge motivation (W) was positioned as a downstream moderator on later-stage links, consistent with the hypothesized pathway-specific role of revenge-related externalization. Specifically, W was allowed to moderate TB → HL (X→M2), PB → HL (M1→M2), and TB → SI (X→Y). For completeness and consistency with the PROCESS-style specification, W was also allowed to moderate the effects of PB and HL on SI (M1→Y and M2→Y), yielding a fully integrated conditional process model.

Formally, the final integrated model (Model 3) can be written as:


$$P\,B=a_{01}+a_{1}\,T\,B+a_{3\,A}\,A\,M+a_{3\,S}\,S\,F+a_{4\,A}\,\left(T\,B\times A\,M\right)+a_{4\,S}\,\left(T\,B\times S\,F\right)+\varepsilon_{1}.$$

$$H\,L=a_{02}+a_{2}\,T\,B+a_{5}\,R\,M+a_{6}\,\left(T\,B\times R\,M\right)+d_{1}\,P\,B+d_{2}\,\left(P\,B\times R\,M\right)+\varepsilon_{2}.$$

$$S\,I=b_{0}+b_{1}\,P\,B+b_{2}\,H\,L+b_{3}\,\left(P\,B\times R\,M\right)+b_{4}\,\left(H\,L\times R\,M\right)+c_{1}\,T\,B+c_{2}\,R\,M+c_{3}\,\left(T\,B\times R\,M\right)+\varepsilon_{3}.$$

#### 2.2.2 Structural plausibility check: serial specification vs. common-cause alternative

Because cross-sectional associations can reflect a shared underlying distress factor rather than a sequential process, we also estimated a theoretically relevant alternative common-cause structure. In this model, TB, PB, and HL were specified as correlated predictors of SI rather than being ordered in a serial chain. We used WAIC/LOOIC to compare the expected out-of-sample predictive performance of these alternative specifications as a structural plausibility check (Vehtari et al., 2017). This comparison evaluates relative predictive adequacy of competing covariance structures and should not be interpreted as evidence of temporal precedence or causal ordering.

#### 2.2.3 Estimation, interactions, and effect summaries

SEMs were estimated using blavaan with the Stan backend (Merkle and Rosseel, 2018). To improve interpretability and reduce nonessential multicollinearity in interaction models, all continuous variables that entered interaction terms were mean-centered prior to constructing product terms (e.g., TB × RM, TB × AM, TB × SF, PB × RM, HL × RM) (Hayes, 2017). Conditional effects were probed at low (-1 SD), mean, and high (+1 SD) levels of each moderator (Hayes, 2017). In particular, to obtain conditional effects for first-stage moderators (AM and SF), we re-centered the moderator to the target level and recomputed the relevant product terms so that the “zero” point in the model corresponded to that moderator level.

Models were fitted via MCMC with 2 chains, 1,000 warmup iterations, and 5,000 post-warmup draws per chain (seed = 1,234). We used Stan control settings adapt_delta = 0.99 and max_treedepth = 15 to reduce divergent transitions and improve sampling stability (Merkle and Rosseel, 2018). Convergence was assessed using rank-normalized R-hat and effective sample size (ESS), using conventional thresholds (R-hat ≤ 1.01; ESS > 400 as a minimum benchmark) (Vehtari et al., 2021). Posterior uncertainty is summarized using 95% credible intervals (CrIs). A complete summary of software versions, MCMC sampling specifications, prior distributions, and convergence criteria for all models is provided in Supplementary Appendix 3.

#### 2.2.4 Model fit, comparison, and inference criteria

To compare SEMs in terms of expected out-of-sample predictive performance, we evaluated models using the Widely Applicable Information Criterion (WAIC) and the Leave-one-out Information Criterion (LOOIC), with lower values indicating better expected predictive performance (Vehtari et al., 2017). Where available, we also reported differences in Expected Log Predictive Density (ΔELPD) together with their associated standard errors (SEs), computed from pointwise PSIS-LOO contributions, to quantify the magnitude and uncertainty of predictive differences between models (Vehtari et al., 2017).

In addition, when computationally feasible, we computed Bayes factors (BFs) based on marginal likelihood evidence, including bridge sampling approaches, to provide complementary evidence for relative model support (Gronau et al., 2020). Convergence and diagnostic checks for PSIS-LOO (e.g., Pareto-k) followed recommended practices (Vehtari et al., 2017).

#### 2.2.5 Stage 2: primary outcome modeling (Bayesian hurdle lognormal model)

Because SI in non-clinical samples often exhibits (a) a large proportion of zeros and (b) right-skew among non-zero values, we modeled SI using a Bayesian hurdle lognormal model (Bürkner, 2017; Hu et al., 2011). Conceptually, the hurdle model separates suicidal ideation into two linked but distinct questions:

1. Presence (threshold) question: “Did any suicidal ideation occur?” (SI > 0 vs. SI = 0).

2. Intensity (severity) question: “If ideation occurred (SI > 0), how severe was it?.”

In brms parameterization, the hurdle (hu) component models the probability of a zero SI score, Pr(SI = 0), via logistic regression (Bürkner, 2017). To make effects easier to interpret in the clinically intuitive “any ideation” direction, we re-expressed hu coefficients as odds ratios for any ideation: OR_*any*_ = exp(-β_hu). Under this reporting convention, OR_*any*_ > 1 indicates increased odds of any suicidal ideation (SI > 0), whereas OR_*any*_ < 1 indicates decreased odds of any suicidal ideation.

The intensity (mu) component models SI severity conditional on SI > 0 using a lognormal regression (Bürkner, 2017; Hu et al., 2011). Effects are summarized as geometric mean ratios (GMRs), where GMR > 1 indicates higher expected SI intensity among those reporting non-zero ideation, and GMR < 1 indicates lower expected intensity.

#### 2.2.6 Hurdle model priors (brms)

To assess the associations between coping moderators and suicidal ideation while accounting for zero-inflation and skew, we specified a single adjusted Bayesian hurdle lognormal model in brms (Bürkner, 2017). Both the hurdle (presence) and intensity (severity) components included the ITS core variables (TB, PB, HL), the coping moderators (RM, AM, SF), and the key interaction terms between TB and each moderator (TB × RM, TB × AM, TB × SF). To reduce scaling artifacts and improve sampling stability, all continuous predictors were mean centered, such that odds ratios and GMRs reflect a one standard deviation change in predictors (Bürkner, 2017). Categorical covariates (gender, education, religion, parental marital status) were modeled as factors, whereas age was modeled as continuous covariates and family income were modeled as categorical factors.

Models were estimated using 4 MCMC chains with 4,000 iterations per chain (1,000 warmup), with a seed of 1,234 and Stan control settings adapt_delta = 0.99 and max_treedepth = 15 to ensure robust sampling (Bürkner, 2017). We employed weakly informative priors suitable for standardized predictors, including normal (0, 1) priors for regression coefficients, a student-t (3, 0, 2.5) prior for intercepts, and a normal (0, 1) prior for the hurdle intercept (Bürkner, 2017). Convergence and sampling adequacy were evaluated using rank-normalized R-hat and effective sample size (ESS) (Vehtari et al., 2021). Posterior summaries are reported as posterior medians with 95% credible intervals (CrIs). For OR_*any*_ and GMR, evidence was considered strong when the 95% CrI excluded the null value (1.00).

#### 2.2.7 Prior sensitivity checks (hurdle model)

Model adequacy was evaluated using posterior predictive checks (pp_check) to assess distributional fit (Gabry et al., 2019). Predictive performance was assessed using PSIS-LOO (LOOIC) and associated Pareto-k diagnostics to identify potentially influential observations; recommended remedial steps (e.g., moment matching) were considered where indicated (Vehtari et al., 2017). To ensure robustness, we conducted a prior sensitivity analysis comparing the default weakly informative specification against a more regularizing prior set. As substantive conclusions remained robust across settings, results are reported under the primary specification, with sensitivity summaries provided in Supplementary Appendix.

#### 2.2.8 Sensitivity to unmeasured confounding (*E*-value)

Given the cross-sectional design, we quantified robustness to potential unmeasured confounding using the *E*-value, defined as the minimum strength of association (on the risk ratio scale) that an unmeasured confounder would need to have with both the exposure and the outcome—conditional on measured covariates—to fully explain away an observed association (VanderWeele and Ding, 2017). *E*-values were computed in R using the *E*-value package (Mathur et al., 2018). Where applicable, *E*-values were reported for both the point estimate and the limit of the 95% interval closest to the null to characterize robustness under conservative assumptions.

## 3 Results

### 3.1 Preliminary analyses

Descriptive statistics and internal consistency reliability for the primary measures are reported in Table 2. Bivariate Spearman correlations are reported in Table 3. Suicidal ideation (SI) was positively associated with thwarted belongingness (TB), perceived burdensomeness (PB), hopelessness (HL), self-dysfunction (SF), and revenge motivation (RM). Associations between the ITS core constructs were substantial, including TB–PB and PB–HL correlations, consistent with an interpersonal-to-cognitive risk architecture.

**TABLE 2:** Means and standard deviations of scales and sub-scales (*N* = 205).

Measure	Mean	SD	Cronbach’s alpha
Transgression-related interpersonal motivations inventory	3.16	0.89	0.87
Revenge motivation	2.40	1.35	0.92
Avoidance motivation	3.71	0.95	0.88
Hopelessness from the beck hopelessness scale	1.96	0.59	0.92
The self and interpersonal functioning scale			
Self-dysfunction	1.81	0.52	0.72
Interpersonal needs questionnaire	2.77	1.03	0.92
Perceived burdensomeness	2.17	1.23	0.93
Thwarted belongingness	3.17	1.11	0.89
The beck scale for suicide ideation (sum score)	2.63	2.67	0.84

**TABLE 3:** Correlations table.

Variable	1	2	3	4	5	6	7
1	–	–	–	–	–	–	–
2	0.405***						
3	0.519***	0.619***					
4	0.470***	0.543***	0.641***				
5	0.088	-0.103	0.038	0.058			
6	0.189**	0.082	0.131	0.223***	0.216**		
7	0.495***	0.560***	0.623***	0.588***	0.049	0.220**	

***p < 0.001, **p < 0.01. *N* = 205. 1 = Suicidal Ideation, 2 = Thwarted Belongingness, 3 = Perceived Burdensomeness, 4 = Hopelessness, 5 = Avoidance Motivation, 6 = Revenge Motivation, 7 = Self-Dysfunction.

### 3.2 Bayesian SEM: structural model testing and model selection

#### 3.2.1 Model comparison (models 1–3 and common cause)

Bayesian SEM model comparison results are summarized in Table 4. Across competing specifications, the integrated model (Model 3) demonstrated the best expected out-of-sample predictive performance. Specifically, Model 3 yielded the lowest information criteria (WAIC = 1673.41; LOOIC = 1674.80). Relative to Model 1, Model 3 showed improved expected predictive accuracy (ΔELPD = 18.42, SE = 7.11) and was favored by marginal likelihood evidence (log-BF = 12.08, favoring Model 3). Model 3 also substantially outperformed Model 2 (ΔELPD = 316.76, SE = 13.83; log-BF = 294.52).

**TABLE 4:** Model fit and information criteria for Bayesian SEM model comparison.

Model comparison	WAIC (A)	WAIC (B)	LOOIC (A)	LOOIC (B)	ELPD diff (SE)	log-BF	Better model
Model 1 vs. model 2	1711.07	2308.14	1711.68	2308.38	298.34 (15.71)	282.44	Model 1
Model 1 vs. model 3	1711.07	1673.41	1711.68	1674.8	–18.42 (7.11)	–12.08	Model 3
Model 2 vs. model 3	2308.14	1673.41	2308.38	1674.8	–316.76 (13.83)	–294.52	Model 3
Common cause vs. model 3	2354.13	1673.41	2354.31	1674.8	–339.74 (17.55)	–301.01	Model 3

WAIC, Widely Applicable Information Criterion; LOOIC, Leave-One-Out Information Criterion; ELPD diff, difference in expected log predictive density (with standard error in parentheses); log-BF, log Bayes factor. Lower WAIC/LOOIC values indicate better model fit.

Critically, Model 3 also outperformed the common-cause alternative. As shown in Table 4, Model 3 yielded substantially lower information criteria (WAIC = 1673.41; LOOIC = 1674.80) than the common-cause model (WAIC = 2354.13; LOOIC = 2354.31), with a large ELPD advantage for Model 3 (Δ ELPD = 339.74, SE = 17.55) and strong marginal likelihood support (log-BF = 301.01, favoring Model 3). Together, these comparisons favored a sequential ITS-consistent specification over a common-cause alternative in this dataset, in terms of expected out-of-sample predictive performance. These model comparisons address relative predictive fit of competing covariance structures and do not establish temporal precedence or causal ordering.

#### 3.2.2 Path coefficients and mediation (model 3)

Parameter estimates for Model 3 are reported in Table 5. Model 3 explained substantial variance in the endogenous variables: *R*^2^ = 0.479 for perceived burdensomeness (PB), *R*^2^ = 0.494 for hopelessness (HL), and *R*^2^ = 0.416 for suicidal ideation (SI).

**TABLE 5:** Comparison of posterior estimates for path coefficients and explained variance across com-peting models.

Predictor path		Model 1 (baseline)		Model 2 (moderated direct)		Model 3 (final comprehensive)			
Outcome	Predictor	*b*	β	*b*	β	*b*	β	Posterior SD	95% Credible interval
Predicting perceived burdensomeness (PB)									
TB		0.639	0.568	0.421	0.408	0.421	0.374	0.068	[0.288, 0.556]
AM		–	–	0.098	0.081	0.098	0.075	0.066	[–0.032, 0.227]
SF		–	–	0.945	0.426	0.946	0.394	0.144	[0.667, 1.229]
RM		0.131	0.142	–	–	–	–	–	–
TB × AM		–	–	0.118	0.131	0.118	0.122	0.050	[0.023, 0.216]
TB × SF		–	–	0.236	0.119	0.235	0.109	0.109	[0.017, 0.450]
TB × RM		0.106	0.136	–	–	–	–	–	–
Predicting hopelessness (HL)									
TB		0.135	0.247	0.119	0.233	0.135	0.249	0.033	[0.070, 0.201]
PB		0.241	0.499	0.254	0.512	0.242	0.501	0.031	[0.182, 0.301]
RM		0.060	0.134	–	–	0.059	0.133	0.023	[0.014, 0.103]
TB × RM		0.048	0.127	–	–	0.048	0.126	0.023	[0.003, 0.092]
PB × RM		–0.047	–0.124	–	–	–0.046	–0.123	0.023	[–0.092, –0.001]
Predicting suicidal ideation (SI)									
TB		–0.070	–0.029	–0.002	–0.001	–0.074	–0.030	0.169	[–0.407, 0.258]
PB		1.053	0.485	1.084	0.477	1.054	0.484	0.177	[0.715, 1.404]
HL		0.842	0.188	0.773	0.169	0.846	0.188	0.348	[0.167, 1.524]
RM		0.145	0.073	–	–	0.146	0.073	0.112	[–0.071, 0.364]
TB × RM		–0.332	–0.196	–	–	–0.333	–0.196	0.113	[–0.555, –0.114]
PB × RM		0.179	0.106	–	–	0.178	0.105	0.138	[–0.095, 0.447]
HL × RM		0.187	0.057	–	–	0.190	0.058	0.250	[–0.302, 0.679]
Explained variance (*R*^2^)									
PB		0.367	–	0.390	–	0.479	–	–	–
HL		0.498	–	0.413	–	0.494	–	–	–
SI		0.412	–	0.353	–	0.416	–	–	–

*b*, Unstandardized Coefficient; β, Standardized Coefficient; Posterior; SD, Posterior Standard Deviation; 95% CrI, 95% Credible Interval. PB, Perceived Burdensomeness; TB, Thwarted Belongingness; HL, Hopelessness; SI, Suicidal Ideation; RM, Revenge Motivation; AM, Avoidance Motivation; SF, Self-Dysfunction. Paths not included in a given model are indicated with a dash (–). Model 3, as the final selected model, includes full posterior statistics. All priors were normal (0, 10). *R*^2^ values represent the proportion of variance explained in each endogenous variable.

Consistent with the hypothesized staged pathways, TB positively associated with PB [*b* = 0.421, 95% CrI (0.288, 0.556)] and HL [*b* = 0.135, 95% CrI (0.070, 0.201)]. PB positively associated with HL [b = 0.242, 95% CrI (0.182, 0.301)] and SI [*b* = 1.054, 95% CrI (0.715, 1.404)]. HL also positively associated with SI [*b* = 0.846, 95% CrI (0.167, 1.524)]. Collectively, these associations were compatible with the specified interpersonal-to-cognitive serial pathway (TB→PB→HL→SI) in this cross-sectional sample.

**FIGURE 4:**
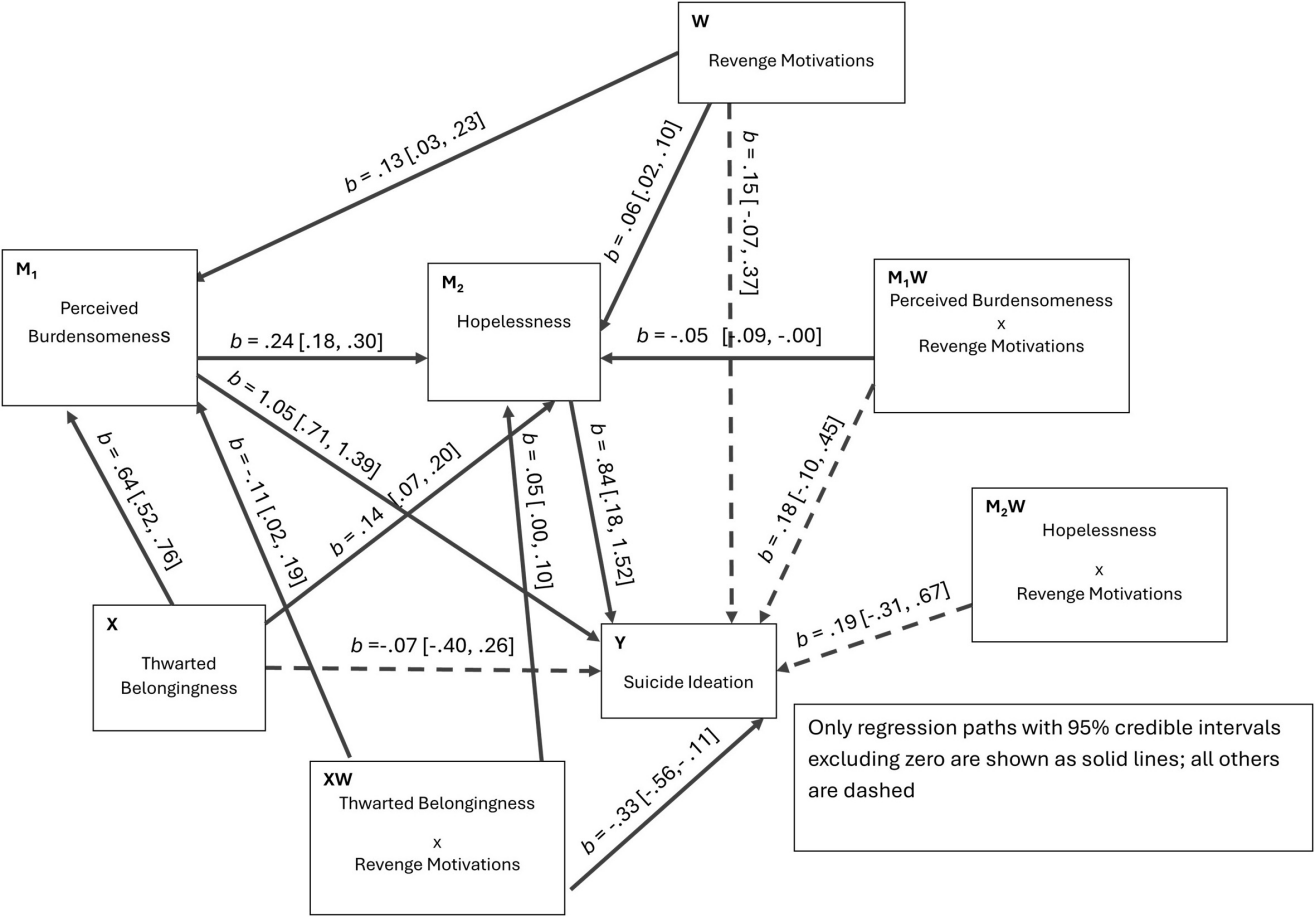
Estimated path diagram for Model 1 (single-moderator serial mediation). Unstandardized regression coefficients (b) with 95% credible intervals are shown. Solid lines indicate statistically significant paths (95% CrI excluding zero); dashed lines indicate non-significant paths.

**FIGURE 5:**
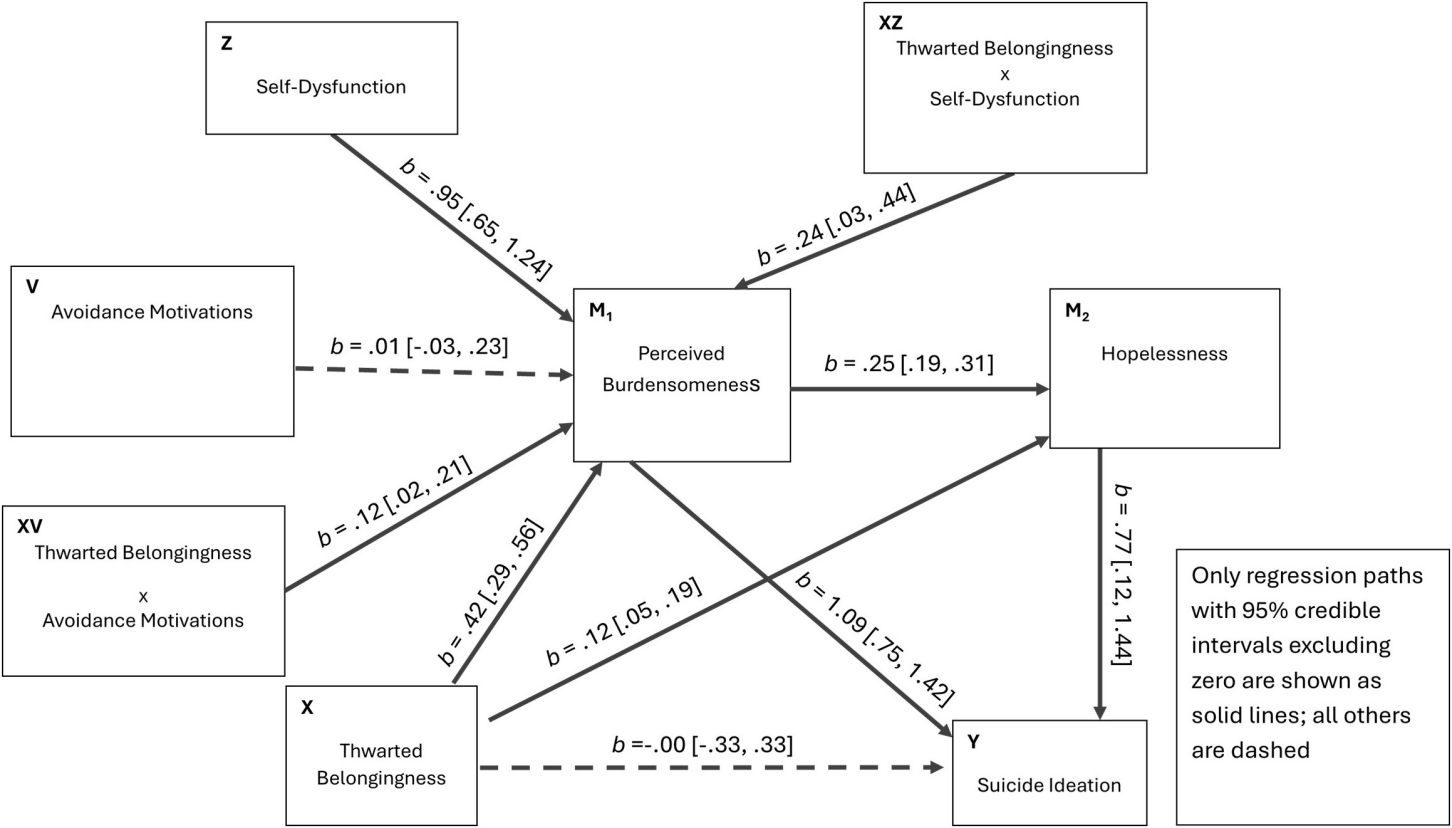
Estimated path diagram for Model 2 (dual first-stage moderation). Unstandardized regression coefficients (b) with 95% credible intervals are shown. Solid lines indicate statistically significant paths; dashed lines indicate non-significant paths.

**FIGURE 6:**
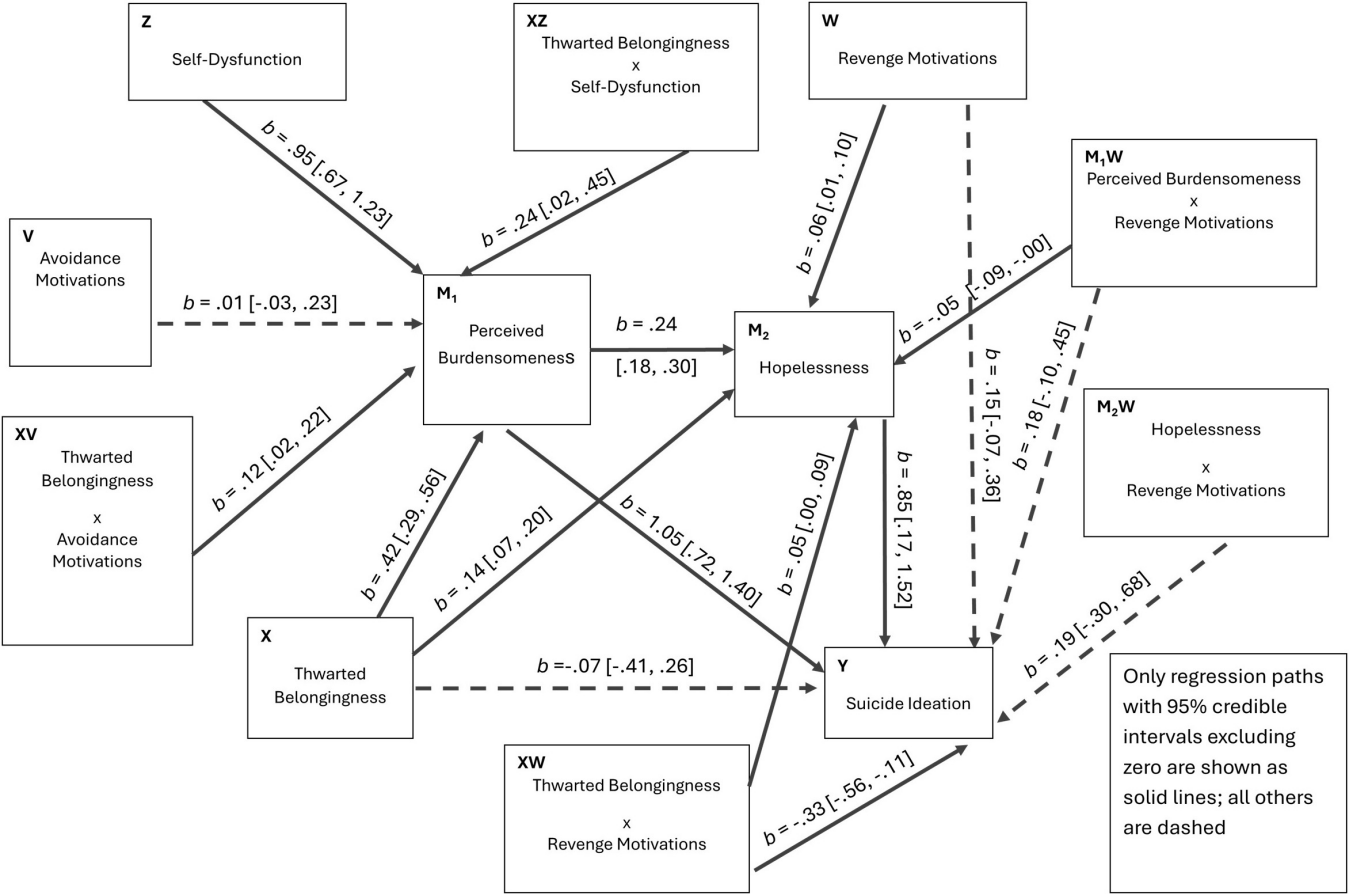
Estimated path diagram for Model 3 (integrated multi-node moderation; final model). Unstandardized regression coefficients (b) with 95% credible intervals are shown. Solid lines indicate statistically significant paths; dashed lines indicate non-significant paths.

For interpretive clarity, Figures 4–6 present the three Bayesian SEM specifications (Models 1–3) in path-diagram form with path estimates. Figures 1–3 are retained as conceptual diagrams corresponding to the hypothesized models, whereas Figures 4–6 display the estimated coefficients.

#### 3.2.3 Mediation effects (model 3)

Defined indirect effects from Model 3 are summarized in Table 6. All three focal indirect effects were supported, including the TB → PB → SI pathway [*b* = 0.444, 95% CrI (0.239, 0.648)], the TB → HL → SI pathway [*b* = 0.114, 95% CrI (0.004, 0.225)], and the serial indirect pathway TB → PB → HL → SI [*b* = 0.086, 95% CrI (0.006, 0.166)]. The direct TB→SI effect at the reference moderator value was not credibly different from zero [*b* = -0.074, 95% CrI (-0.405, 0.257)], whereas the total effect was positive and credibly different from zero [*b* = 0.570, 95% CrI (0.250, 0.889)]. Together, this pattern is consistent with a primarily indirect pathway from thwarted belongingness to suicidal ideation through perceived burdensomeness and hopelessness.

**TABLE 6:** Indirect, direct, and total effects of thwarted belongingness on suicidal ideation (model 3).

Effect path	Posterior mean	95% Credible interval	β (Std.all)
Total indirect effect	0.644	[0.350, 0.980]	
Specific indirect 1: TB → PB → SI	0.444	[0.239, 0.648]	0.181
Specific indirect 2: TB → HL → SI	0.114	[0.004, 0.225]	0.047
Serial indirect: TB → PB → HL → SI	0.086	[0.006, 0.166]	0.035
Direct: TB → SI	–0.074	[–0.405, 0.257]	-0.030
Total effect	0.570	[0.250, 0.889]	0.232

PB, Perceived Burdensomeness; TB, Thwarted Belongingness; HL, Hopelessness; SI, Suicidal Ideation.

#### 3.2.4 Node-specific moderation effects (model 3)

Model 3 supported the hypothesized node-specific moderation pattern (Table 5). At the first stage, interaction terms indicated that the TB–PB association was stronger at higher levels of avoidance motivation and self-dysfunction, as indicated by positive interaction effects for TB × AM [*b* = 0.118, 95% CrI (0.023, 0.216)] and TB × SF [*b* = 0.235, 95% CrI (0.017, 0.450)]. Downstream, revenge motivation showed differentiated pathway-specific effects. RM was associated with a stronger TB–HL association (positive TB × RM) and a weaker PB–HL association (negative PB × RM) in the specified model [PB × RM; *b* = -0.046, 95% CrI (-0.092, -0.001)]. RM also moderated the direct TB → SI path [TB × RM; *b* = -0.333, 95% CrI (-0.555, -0.114)], indicating that higher revenge motivation was associated with a weaker (more negative) direct TB–SI association in this model.

**FIGURE 7:**
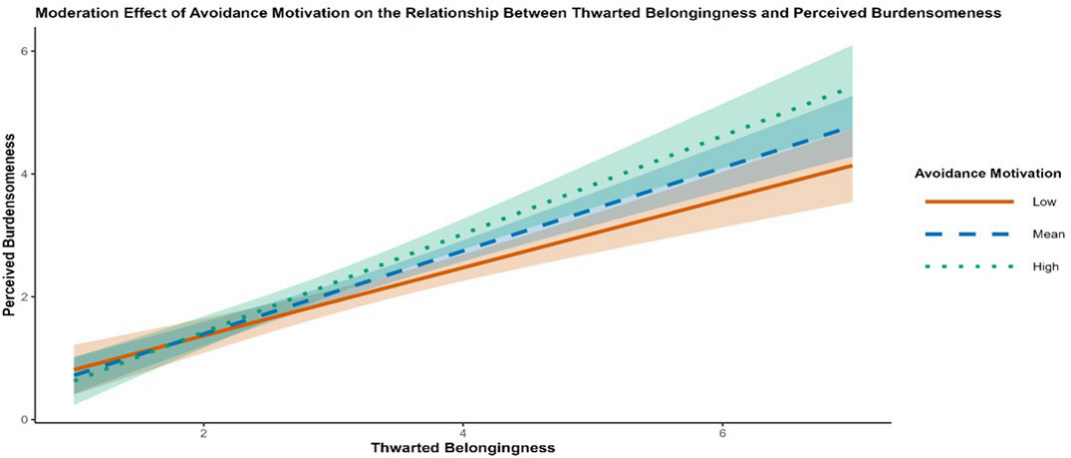
Simple slopes plot showing the moderation effect of avoidance motivation on the association between thwarted belongingness and perceived burdensomeness. Lines represent the conditional association at low (–1 SD; solid orange), mean (dashed blue), and high (+1 SD; dotted green) levels of avoidance motivation. Shaded regions indicate 95% credible intervals.

**FIGURE 8:**
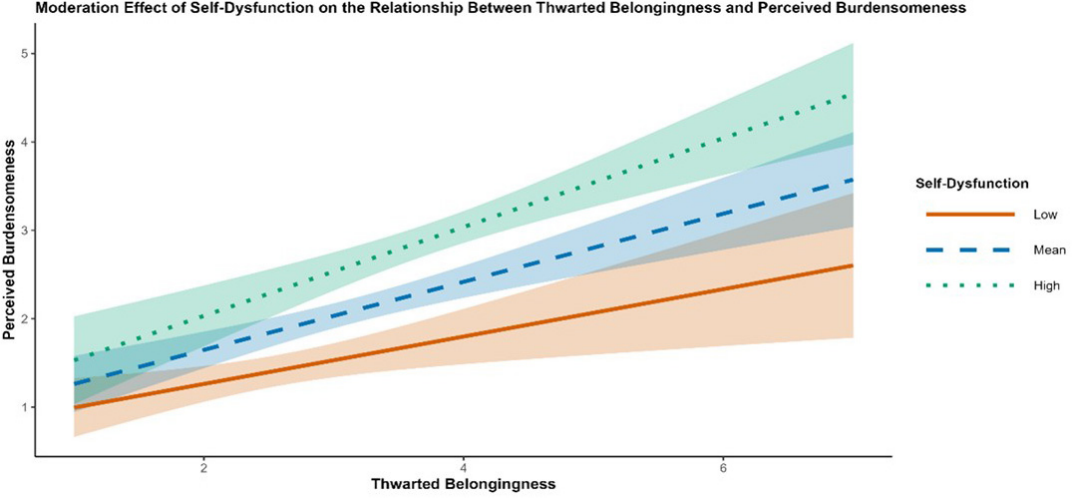
Simple slopes plot showing the moderation effect of self-dysfunction on the association between thwarted belongingness and perceived burdensomeness. Lines represent the conditional association at low (–1 SD), mean, and high (+1 SD) levels of self-dysfunction. Shaded regions indicate 95% credible intervals.

**FIGURE 9:**
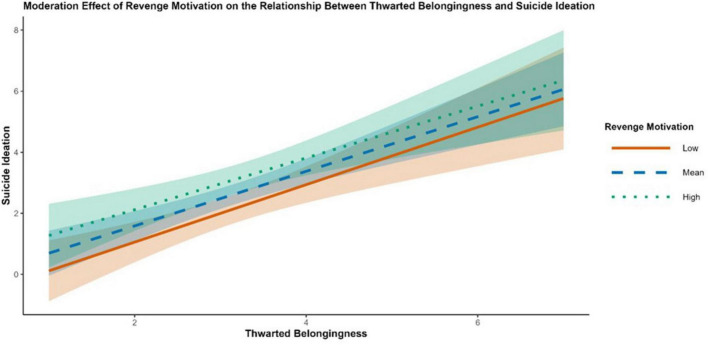
Simple slopes plot showing the moderation effect of revenge motivation on the association between thwarted belongingness and suicidal ideation. Lines represent the conditional association at low (–1 SD), mean, and high (+1 SD) levels of revenge motivation. Shaded regions indicate 95% credible intervals.

Conditional effects at low (-1 SD), mean, and high (+1 SD) levels of each moderator are summarized in Table 7. Consistent with first-stage moderation, the indirect effect TB → PB → SI increased as AM and SF increased [e.g., AM low: *b* = 0.326, 95% CrI (0.129, 0.523); AM high: *b* = 0.563, 95% CrI (0.303, 0.823); SF low:.316 95% CrI (0.094, 0.538); SF high: *b* = 0.574 (0.312, 0.836)]. Conditional estimates for RM further indicated that the direct TB → SI pathway varied across RM levels. The association was not credibly different from zero at low RM [direct effect *b* = 0.377, 95% CrI (-0.028, 0.782)] but became credibly negative at high RM [direct effect b = -0.525, 95% CrI (-1.009, -0.041)]. Figures 7, 8 visualize the first-stage interactions via simple slopes plots, showing steeper TB → PB associations at higher AM and higher SF. Figure 9 presents the corresponding moderation effect of revenge motivation on the direct TB → SI association, illustrating the attenuation of this path at higher levels of revenge motivation.

**TABLE 7:** Conditional direct and indirect effects across levels of moderators (model 3).

Path	Moderator level	Level	Indirect effect *b*	SD	95% Credible interval
Effects on perceived burdensomeness (PB)					
TB → PB	Avoidance motivation (AM)	Low (-1 SD)	0.554	0.078	[0.401, 0.708]
		Mean	0.676	0.063	[0.553, 0.800]
		High (+1 SD)	0.798	0.084	[0.633, 0.964]
	Self-dysfunction (SF)	Low (-1 SD)	0.268	0.092	[0.088, 0.448]
		Mean	0.385	0.068	[0.251, 0.519]
		High (+1 SD)	0.502	0.086	[0.334, 0.671]
Effects on hopelessness (HL)					
TB → HL	Revenge motivation (RM)	Low (-1 SD)	0.214	0.042	[0.132, 0.296]
		Mean	0.279	0.031	[0.219, 0.339]
		High (+1 SD)	0.343	0.042	[0.260, 0.425]
PB → HL	Revenge motivation (RM)	Low (-1 SD)	0.340	0.043	[0.256, 0.423]
		Mean	0.312	0.026	[0.260, 0.364]
		High (+1 SD)	0.284	0.032	[0.221, 0.348]
Effects on suicide ideation (SI)					
TB → SI	Revenge motivation (RM)	Low (-1 SD)	0.377	0.207	[–0.028, 0.782]
		Mean	–0.074	0.169	[–0.405, 0.257]
		High (+1 SD)	–0.525	0.247	[–1.009, –0.041]
TB → PB → SI	Avoidance motivation (AM)	Low (-1 SD)	0.326	0.101	[0.129, 0.523]
		Mean	0.444	0.104	[0.239, 0.648]
		High (+1 SD)	0.563	0.133	[0.303, 0.823]
	Self-dysfunction (SF)	Low (-1 SD)	0.316	0.113	[0.094, 0.538]
		Mean	0.444	0.104	[0.239, 0.648]
		High (+1 SD)	0.574	0.134	[0.312, 0.836]
	Revenge motivation (RM)	Low (-1 SD)	0.342	0.135	[0.077, 0.607]
		Mean	0.444	0.104	[0.239, 0.648]
		High (+1 SD)	0.545	0.129	[0.292, 0.798]

*b*, Unstandardized posterior mean estimate; SD, Posterior Standard Deviation; CrI, Credible Interval. TB, Thwarted Belongingness; PB, Perceived Burdensomeness; HL, Hopelessness; SI, Suicidal Ideation; AM, Avoidance Motivation; SF, Self-Dysfunction; RM, Revenge Motivation. The table summarizes the simple slopes for specific paths identified as significant in the node-specific moderation analysis.

### 3.3 Bayesian hurdle lognormal model (adjusted): associations with ideation presence and severity

To obtain distribution-appropriate estimates for suicidal ideation in the presence of excess zeros and skew, we fitted an adjusted Bayesian hurdle lognormal model (Table 8). Convergence diagnostics were excellent (R-hat approximately 1.00; large effective sample sizes).

**TABLE 8:** Adjusted bayesian hurdle lognormal model predicting suicidal ideation.

Predictor	Hurdle component (presence of ideation)		Intensity component (severity of ideation)	
	Odds ratio (OR_any_; any ideation)	95% Credible interval	Geo. mean ratio (GMR)	95% Credible interval
ITS Main components				
TB	1.32	[0.77, 2.31]	1.01	[0.89, 1.15]
PB	1.93*	[1.07, 3.71]	1.19*	[1.07, 1.33]
HL	1.90	[0.65, 5.83]	1.19	[0.92, 1.53]
Moderators				
RM	1.01	[0.69, 1.45]	0.98	[0.90, 1.07]
AM	1.04	[0.64, 1.68]	1.1	[0.96, 1.26]
SF	2.36	[0.75, 7.82]	1.18	[0.91, 1.52]
Interactions				
TB × RM	0.51*	[0.34, 0.72]	1.03	[0.95, 1.12]
TB × AM	1.27	[0.78, 2.01]	0.93	[0.85, 1.02]
TB × SF	1.4	[0.61, 3.46]	0.96	[0.80, 1.14]
Control variables (selected)				
Age	0.98	[0.86, 1.10]	0.97	[0.94, 1.00]
Gender	2.68*	[1.05, 7.26]	0.85	[0.67, 1.10]

OR, Odds Ratio; GMR, Geometric Mean Ratio. Bold* indicates 95% Credible Interval excludes 1 (significant). Model adjusted for Age, Gender, Religion, Family Income, Education, and Parental Marital Status. PB, Perceived Burdensomeness; TB, Thwarted Belongingness; HL, Hopelessness; SI, Suicidal Ideation; RM, Revenge Motivation; AM, Avoidance Motivation; SF, Self-Dysfunction.

#### 3.3.1 Hurdle component (any ideation vs. none)

Perceived burdensomeness was strongly associated with the odds of reporting any suicidal ideation, OR_any_ = 1.93, 95% CrI [1.07, 3.71]. Gender (male vs. female) was also associated with higher odds of any ideation, OR_any_ = 2.68, 95% CrI [1.05, 7.26].

A key interaction emerged for TB × RM in the hurdle component: OR_any_ = 0.51, 95% CrI [0.34, 0.72], indicating that higher RM attenuated the TB–ideation association in terms of crossing the threshold from no ideation to any ideation.

#### 3.3.2 Intensity component (severity among non-zero ideation)

Among participants with non-zero ideation, perceived burdensomeness was again associated with greater ideation intensity, GMR = 1.19, 95% CrI [1.07, 1.33]. Other main effects and interactions did not show credible departures from the null in the adjusted intensity component. Complete parameter estimates for all predictors and covariates (including categorical specifications for income and socio-demographics), along with model convergence diagnostics, are provided in Supplementary Appendix 2. Convergence diagnostics for all interaction terms were satisfactory (R-hat ≤ 1.01; ESS > 400 for all parameters; see Supplementary Appendix 2 for full diagnostics). However, 95% credible intervals for several interaction coefficients were moderately wide [e.g., TB × SF: (0.017, 0.450); TB × RM on the direct path: (-0.555, -0.114)], consistent with the exploratory nature of these conditional effects and the modest sample size. Visual inspection of trace plots and posterior density plots confirmed that posterior distributions for all interaction parameters were unimodal and well-mixing, with no evidence of multimodality or chain non-convergence (see Supplementary Appendix 2 for full diagnostics). These estimates should therefore be interpreted as preliminary evidence of node-specific moderation patterns warranting replication in larger samples.

### 3.4 Prior sensitivity and prior predictive checks (summary)

Prior predictive variance summaries for the default versus more regularizing prior sets are reported in Supplementary Table 1 and visualized in Supplementary Figures 1–6. Under the default weakly informative priors, the prior predictive distribution allowed very wide outcome variability, whereas the more regularizing priors yielded substantially narrower prior predictive variance. Conclusions in the primary models were robust to this sensitivity check; therefore, results are reported under the default prior specification.

### 3.5 Sensitivity to unmeasured confounding (*E*-values)

For the adjusted hurdle model association reported for TB in the “any ideation” framing [OR = 1.321, 95% CrI (0.770, 2.313)], the *E*-value was 1.56 (lower limit = 1.00), indicating that an unmeasured confounder would require a risk ratio association of approximately 1.56 with both exposure and outcome to fully explain away the observed point estimate (VanderWeele and Ding, 2017). *E*-values were computed for the TB main effect (OR_any_) as a parsimonious summary; interaction-specific *E*-values were not computed due to interpretational complexity.

## 4 Discussion

Grounded in the Interpersonal Theory of Suicide (ITS) (Van Orden et al., 2010), our results were consistent with a pattern in which thwarted belongingness (TB) was associated with perceived burdensomeness (PB), PB was associated with hopelessness (HL) and suicidal ideation (SI); and HL was associated with SI. By employing a Bayesian hurdle modeling approach, we further clarified that perceived burdensomeness is robustly associated with both the probability of having any suicidal ideation and the intensity of those thoughts among those who report them. Beyond these direct links, unforgiveness-related coping tendencies exhibited “node-specific” moderating effects: avoidance and self-dysfunction were associated with a stronger TB–PB association (i.e., positive interaction terms), while revenge motivation showed a differentiated pattern (stronger TB–HL association alongside lower odds of reporting any SI), which was interpreted as consistent with outward deflection/externalization rather than durable protection. Collectively, these findings elucidate how different coping styles shape the classic interpersonal pathway from social pain to suicidal thinking (Chu et al., 2017). Because the data are cross-sectional, all “paths” refer to modeled associations; directionality is theory-driven and should be tested prospectively.

### 4.1 Specific mechanisms of moderators

#### 4.1.1 Self-dysfunction: Identity-based amplification of burdensomeness

Identity instability and impaired self-direction—core features of self-dysfunction in the DSM-5-TR Alternative Model for Personality Disorders (American Psychiatric Association [APA], 2022)—may be associated with a greater tendency to interpret interpersonal stressors as evidence of personal deficits, which could coincide with higher perceived burdensomeness (PB). This interpretation aligns with research positioning the level of personality functioning as a transdiagnostic marker of self-other functional impairment relevant to suicide risk (Bender et al., 2011) and with the ITS literature confirming the robust link between PB and SI (Chu et al., 2017; Van Orden et al., 2010). Clinically, interventions aimed at clarifying identity and values, enhancing self-direction, and reducing shame-based self-criticism could be examined as strategies to potentially weaken the TB–PB association and reduce correlated hopelessness/SI. Incorporating elements of self-forgiveness would be particularly beneficial for reducing self-directed shame and punitive self-standards (Hall and Fincham, 2005; Woodyatt et al., 2017).

#### 4.1.2 Avoidance: a withdrawal–defeat maintenance loop

While potentially reducing distress in the short term, an avoidant coping may deprive individuals of corrective social experiences, potentially maintaining the isolation, defeat, and entrapment processes linked to suicidal ideation (Siddaway et al., 2015; Taylor et al., 2011). Meta-analytic evidence further situates avoidance within a broader framework of maladaptive emotion regulation strategies associated with a range of psychopathologies (Aldao et al., 2010). In the present data, results indicated that avoidance amplified the TB → PB path, consistent with interpersonal models of social anxiety showing a self-perpetuating cycle of withdrawal–rejection expectancy (Alden and Taylor, 2004) and with ostracism research highlighting the pain of exclusion (Williams, 2007). Practically, graded social exposure and structured problem-solving may help restore a sense of agency and social efficacy (D’Zurilla and Nezu, 2010), interrupting the withdrawal-defeat loop that entrenches beliefs of burdensomeness and hopelessness.

#### 4.1.3 Revenge motivation: externalization as an unstable deflection

A noteworthy finding from the hurdle model was that higher revenge motivation (RM) was associated with a reduced likelihood of reporting any suicidal ideation (lower OR for the hurdle component), despite amplifying the upstream TB → HL path. An integrative explanation distinguishes between the externalization and internalization of distress. Shame-proneness is linked to angry rumination (Tangney et al., 1996), and anger expression can be directed externally rather than internally (Spielberger, 1999). When RM is high, the psychache (Shneidman, 1996, 1998) stemming from interpersonal stressors may be preferentially externalized onto others rather than internalized as self-attack (i.e., suicidal ideation).

From an emotion-regulation perspective, this pattern may also reflect a distinction between activating and deactivating affective states. Anger—the core emotion underlying revenge motivation—is a high-arousal, approach-oriented affect that mobilizes goal-directed action toward the perceived source of harm (Carver and Harmon-Jones, 2009). By contrast, hopelessness and suicidal ideation are characterized by low arousal, behavioral withdrawal, and the cessation of goal pursuit—affective states more consistent with defeat and entrapment (Taylor et al., 2011). Importantly, the shame–anger transformation may be central to this affective shift. As Katz (1988) observed, “transforming humiliation into rage” (p. 22) represents a rapid emotional escalation from a deactivated state (shame, defeat) to an activated one (anger, approach motivation), and Scheff (1994) further argued that shame–anger sequences function as self-reinforcing “feeling traps” in which shame automatically generates anger that, in turn, intensifies the original shame. Once this transformation occurs, the sustained cognitive engagement required by revenge—which McCullough et al. (2013) characterize as “anger transformed into rumination, which is a highly elaborated cognitive activity that includes detailed (sometimes fanciful) planning for the future” (p. 33)—may further maintain this activated orientation, displacing the passive, defeated posture that characterizes suicidal ideation. This mechanism aligns with the distinction between externalized and internalized psychache (Shneidman, 1996, 1998): when the devalued self is “externalized through projection, others are perceived as defective and inferior, justifying the release of aggression through contempt, envy, and rage” (Hahn, 2000, p. 12), thereby diverting distress from the self-directed pathway that culminates in suicidal ideation (Tangney et al., 1996). However, this displacement is likely to be transient: as retaliatory goals are thwarted or anger dissipates, the underlying despair and hopelessness—which remain elevated, as indicated by the positive TB→HL moderation—may resurface, potentially with increased intensity due to accumulated interpersonal damage (Dollard et al., 1939/2013; O’Connor and Kirtley, 2018).

However, this mechanism should be interpreted as a “deflection” rather than adaptive protection. While externalizing aggression may temporarily mask the link to suicidal ideation, it does not resolve the underlying interpersonal dysfunction. Instead, it creates a “double-edged sword” scenario: the individual may be temporarily shielded from the “I am a burden” cognition by blaming others, but this coping style likely exacerbates interpersonal conflict and isolation in the long run. This interpretation aligns with research suggesting that while externalizing behaviors may inversely correlate with internalizing symptoms like depression and suicidality in the short term, they often coexist with impulsivity and poor psychosocial functioning (Mann et al., 1999; Mann, 2003).

Furthermore, McCullough et al. (1997, 1998) propose that true forgiveness involves decreased retaliation and avoidance motivations coupled with increased conciliation. Our findings are consistent with patterns that may emerge when this process fails: increased revenge and avoidance motivations emerge that, while potentially deflecting against immediate self-harm, may perpetuate the underlying cycle of unforgiveness. Research indicates that genuine healing requires both self-forgiveness and forgiveness of others—processes that actively reduce unforgiveness rather than merely redirecting it (Fisher and Exline, 2010; McGaffin et al., 2013).

Overall, this pattern should not be interpreted as broadly protective. The ITS risk architecture likely remains active, and externalized anger can rapidly turn inward when revenge is thwarted or helplessness increases (Mann et al., 1999; Mann, 2003; Turecki and Brent, 2016). Given the overlapping diatheses for suicidality and aggression—such as impulsivity and impaired decision-making (Mann et al., 1999; Mann, 2003)—revenge motivation may simply divert risk rather than eliminate it.

### 4.2 Theoretical and clinical implications

#### 4.2.1 Novel theoretical contributions

This study advances the literature in three ways. First, it is preliminary cross-sectional evidence that how unforgiveness-coping strategies moderate established interpersonal suicide pathways at specific nodes (via differential effects on early vs. late links; Chu et al., 2017; Van Orden et al., 2010). Second, it characterizes the paradoxical short-term deflection of externalization while maintaining clinical vigilance for its instability and risks (via a potentially reversible, context-dependent buffering; Mann, 2003; Tangney et al., 1996). Third, it offers a unifying “transgression–unforgiveness” framework that bridges the ITS and IMV models with direct clinical utility (via specifically clarifying the paradoxical intervention’s targets and timing; O’Connor and Kirtley, 2018; Wenzel and Beck, 2008).

#### 4.2.2 Connecting ITS to the integrated motivational–volitional model

The IMV model (O’Connor, 2011; O’Connor and Kirtley, 2018) proposes a three-phase process—pre-motivational, motivational, and volitional—in which background factors and triggering events give rise to feelings of defeat and entrapment (motivational phase), which, under certain moderating conditions (e.g., threat-to-self appraisals), transition into suicidal ideation and behavior (volitional phase). Our findings may help bridge the ITS (Van Orden et al., 2010) with O’Connor’s Integrated Motivational-Volitional (IMV) model (O’Connor, 2011; O’Connor and Kirtley, 2018) by specifying how unforgiveness-coping moderates phase transitions. Although defeat and entrapment were not measured directly, the observed moderation patterns align with IMV predictions: avoidance may act as a “Threat-to-Self” moderator, channeling interpersonal defeat into entrapment through withdrawal and ineffective problem-solving (Aldao et al., 2010; Siddaway et al., 2015; Tucker et al., 2016), while TB and PB are consistent with motivational phase mechanisms that enhance the transition from entrapment to ideation by entrenching negative self and social beliefs (Beck et al., 1999; Christensen et al., 2013; Kleiman et al., 2014). Revenge motivation, in turn, appears to direct the course of distress in the motivational phase—outward rather than inward (via anger and retaliatory ideation; Spielberger, 1999; Tangney et al., 1996)—and may influence the volitional phase by altering behavioral output, even while the burdensomeness-hopelessness mechanism persists (by diverting motivation away from self-harm) (Klonsky and May, 2015).

This integration highlights three implications: (a) unforgiveness-coping (avoidance, self-dysfunction, revenge) can serve as coherent moderators within the IMV model’s motivational phase; (b) the quality of interpersonal connection is more critical than mere contact for breaking defeat-entrapment cycles (Baumeister and Leary, 1995; Williams, 2007); and (c) intervention timing is crucial—addressing unforgiveness and coping styles early may prevent individuals from crossing IMV phase thresholds (O’Connor and Kirtley, 2018).

#### 4.2.3 Clinical assessment and intervention implications

Beyond individual care, these modeled correlates could be operationalized in youth-serving systems as brief screening of thwarted belongingness, perceived burdensomeness, hopelessness, and unforgiveness-based coping motives to focus stepped-care prevention and early support (Chu et al., 2017; Turecki and Brent, 2016; Van Orden et al., 2010). To align clinical decision-making with these findings (Chu et al., 2017; Joiner and Rudd, 1995), risk assessment may benefit from incorporating the following dimensions: (a) history of significant transgressions and betrayals; (b) current unforgiveness and coping patterns—avoidance, externalization vs. internalization, and self-dysfunction (McCullough and Hoyt, 2002; Thompson et al., 2005); (c) the presence, content, and controllability of revenge fantasies (Spielberger, 1999); (d) the quality versus quantity of social relationships, including experiences of ostracism and hostile exchanges (Newsom et al., 2008; Rook, 2015; Williams, 2007); (e) feelings of defeat and entrapment (Siddaway et al., 2015; Taylor et al., 2011); and (f) the interaction between revenge motivation and impulsivity/aggression (Mann et al., 1999; Mann, 2003).

These insights suggest that suicide prevention efforts could be strengthened by moving beyond symptom management to address the underlying unforgiveness cycle. Rather than viewing revenge motivation as purely problematic or purely protective, clinicians might acknowledge its role as a defense mechanism against shame and internal pain. This might involve helping clients transition from externalized aggression (revenge) and withdrawal (avoidance) toward more adaptive coping that includes self-forgiveness and, when appropriate and safe, forgiveness of others. Boundary conditions and population-specific risks are detailed in the limitations. At the population level, programs that strengthen social connectedness and reduce hostile exchanges may mitigate upstream interpersonal risk, while anger regulation combined with self-/other-forgiveness work addresses unforgiveness-maintained psychache; such strategies align with evidence linking defeat/entrapment, emotion regulation, and ostracism to suicidal ideation (Aldao et al., 2010; Siddaway et al., 2015; Taylor et al., 2011; Wade et al., 2014; Williams, 2007).

#### 4.2.4 Public health implications

Suicide among young people is a priority public health concern and a significant source of preventable mortality worldwide (Lim et al., 2019; Mortier et al., 2018; Turecki and Brent, 2016). Our findings inform health promotion and disease prevention at the population level by specifying upstream mechanisms that can be addressed before suicidal ideation emerges or escalates. Routine, brief screening in youth-serving settings (e.g., universities, community health centers) could be used to assess the psychosocial determinants mapped in this model—thwarted belongingness, perceived burdensomeness, hopelessness, and unforgiveness-based coping—enabling the identification of a broader at-risk group to guide stepped-care responses (Chu et al., 2017; Van Orden et al., 2010).

Targeted, scalable prevention can then be tailored to pathway-specific needs. For instance, campus-wide social reconnection initiatives can mitigate feelings of thwarted belonging. At the same time, digital or group-based modules can weaken the TB→PB link by addressing shame-laden burden beliefs through interventions focused on identity, self-direction, and self-forgiveness (Hall and Fincham, 2005; McGaffin et al., 2013; Thompson et al., 2005), as well as the defeat and entrapment processes implicated in escalation (Alden and Taylor, 2004; Siddaway et al., 2015; Taylor et al., 2011; Williams, 2007). Findings on revenge motivation translate into a key public health message: externalizing anger is an unstable buffer. Public health guidance may therefore promote holistic strategies—pairing anger-regulation skills with forgiveness-based approaches, connection-building, and burden-belief reduction—rather than relying on externalization alone (Turecki and Brent, 2016; Wade et al., 2014).

For policymakers, these findings suggest the value of prioritizing funding for low-intensity, community-based programs that foster forgiveness and social efficacy, with the potential to reduce ideation risk among high-risk youth. For researchers, this work provides a foundation for testing cultural adaptations in collectivistic contexts (e.g., Hong Kong), where family harmony shapes dynamics of unforgiveness (Bedford and Yeh, 2019; Yeh and Bedford, 2003). Together, these multi-level strategies target modifiable social and mental environmental determinants of suicidal ideation in youth, offering tangible benefits for wellbeing and quality of life.

### 4.3 Limitations, cultural context, and future directions

Several limitations warrant caution. First and foremost, the cross-sectional design precludes any causal inference. While our model comparison supports the statistical plausibility of the serial sequence, we cannot rule out bidirectional effects or concurrent operation of the variables. Terms such as “pathway” or “mechanism” refer to statistical associations within the modeled framework, not to established temporal causality. Second, the modest, predominantly female (72.2%), non-clinical university sample constrains external validity. The low base rate of suicidal ideation in this community sample, while appropriately handled by the hurdle model, means that the adequate sample size for the intensity component (i.e., participants reporting SI > 0) was limited, reducing precision for severity-specific estimates and potentially inflating standard errors for interaction terms in this component. These findings may not capture risk processes operating in clinical or acutely distressed populations. Third, internal consistency for the Self-Dysfunction subscale was acceptable but modest. Fourth, several theoretically important constructs were not directly measured, including defeat and entrapment, trait impulsivity, aggression proneness, acquired capability for suicide, the direction of anger (internalization versus externalization), and depressive symptoms. Although hopelessness—a core component of depression and a proximal predictor in the ITS model—was controlled for, and sensitivity analyses (*E*-values) suggest robustness to unmeasured confounding, the omission of a broad measure of depression remains a limitation. Fifth, it is possible that the observed moderation pattern for revenge motivation partly reflects characteristics of the specific items used, which assess retaliatory intent rather than enacted aggression; future work should examine whether this pattern replicates with behavioral measures of revenge or alternative operationalizations of externalized coping. Collectively, the absence of these variables limits mechanistic precision and the ability to model phase transitions (Joiner, 2005; Mann et al., 1999; Mann, 2003; O’Connor and Kirtley, 2018; Siddaway et al., 2015; Spielberger, 1999; Taylor et al., 2011).

#### 4.3.1 Boundary conditions and high-risk contexts

The apparent deflection associated with revenge motivation should be treated as conditional and potentially unstable. When retaliatory aims are blocked or thwarted, outwardly directed anger may invert into self-directed despair, with concomitant increases in entrapment and hopelessness—patterns consistent with frustration–aggression dynamics and motivational accounts of entrapment (Dollard et al., 1939/2013; O’Connor and Kirtley, 2018; Taylor et al., 2011). Coupling high revenge motivation with diatheses such as impulsivity or impaired decision-making—traits linked to both suicidality and aggression—may shift externalization from a transient diversion of distress to a pathway toward harmful action; repetitive rehearsal of revenge may also increase acquired capability for suicide through habituation to pain and death (Joiner, 2005; Mann et al., 1999; Mann, 2003). Generalizability is further bounded by population characteristics: effects observed in community-dwelling young adults may not extend to more severely traumatized or clinically complex groups. In presentations marked by affective instability and impulsive aggression (e.g., borderline personality disorder) or post-traumatic hyperreactivity (e.g., PTSD), revenge-related processes may exacerbate dysregulation and self-injury rather than attenuate ideation (Linehan, 1993; Paris, 2002; Turecki and Brent, 2016).

#### 4.3.2 Cultural context

The cultural context of Hong Kong is also relevant to interpreting these findings. In Chinese societies, social harmony and the preservation of “face” (mianzi) are paramount (Ho, 1976). Interpersonal transgressions can induce profound shame, which is often managed through covert strategies to avoid public confrontation. In this context, the expression of revenge may manifest as a private fantasy rather than overt aggression, serving as a cognitive strategy to restore a sense of power without publicly disrupting social harmony (Bedford and Yeh, 2019; Ho, 1976). This cultural nuance might explain why revenge motivation appeared to deflect internalizing symptoms (suicidal ideation) in our sample—it offers a psychological exit from the shame of “losing face” without necessarily leading to immediate behavioral consequences. By contrast, in the Western samples where the ITS was primarily developed and tested, revenge motivation has typically been associated with externalizing psychopathology and aggression rather than examined as a moderator of internalizing pathways (Mann et al., 1999; Mann, 2003). The present findings raise the possibility that the function of revenge motivation within ITS pathways may be partly culturally shaped, operating as covert cognitive restoration of self-worth in face-oriented societies rather than as overt behavioral retaliation.

Additionally, Yeh and Bedford’s (2003) Dual Filial Piety model distinguishes between reciprocal filial piety—grounded in emotional closeness and gratitude—and authoritarian filial piety, which demands compliance with parental authority and the suppression of personal grievances for the sake of family harmony. Under authoritarian filial piety, individuals who experience transgressions by family elders may be culturally constrained from expressing anger or seeking direct confrontation, potentially channeling unforgiveness inward, thereby sustaining identity-related shame and reinforcing perceived burdensomeness (Bedford and Yeh, 2019). This cultural dynamic may be particularly relevant to the present findings, as it suggests that the avoidance and self-dysfunction pathways observed in our model may be amplified in contexts where direct expression of interpersonal grievances carries high relational costs. Consequently, interventions that emphasize self-forgiveness and internal healing without requiring forced reconciliation may be especially appropriate in such settings (Bedford and Yeh, 2019; Yeh and Bedford, 2003). Finally, it is important to note that the premise of perceived burdensomeness may not encompass all forms of self-destructive behavior in this cultural milieu; altruistic suicide and strategic, group-embedded self-sacrifice follow distinct motivational logics that diverge from the belief “I am a burden” (Durkheim, 2002; Leenaars, 2004; Yeh and Bedford, 2003).

#### 4.3.3 Future directions

Prospective studies should evaluate these boundary conditions by directly measuring defeat and entrapment, impulsivity and aggression proneness, anger directionality, and acquired capability, and by modeling context-dependent shifts between externalization and internalization (Joiner, 2005; Klonsky and May, 2015; O’Connor and Kirtley, 2018; Siddaway et al., 2015; Taylor et al., 2011). In particular, future longitudinal designs are warranted to support the hypothesized sequencing and test whether the moderation dynamics observed in this cross-sectional analysis hold when temporal ordering can be established. Intervention trials could test whether forgiveness-based approaches, supplemented with graded social exposure and structured problem solving, reduce unforgiveness-maintained risk more effectively than symptom-focused care (D’Zurilla and Nezu, 2010; Enright and Fitzgibbons, 2000; Wade et al., 2014). Replication in higher-risk clinical samples is essential to establish external validity and population-specific boundary conditions (Turecki and Brent, 2016).

## 5 Conclusion

This study describes an ITS-informed staged interpersonal-to-cognitive ordering of correlates of suicidal ideation. Using a hurdle model, we found that perceived burdensomeness was a robust correlate of both the emergence and intensity of suicidal thoughts. We examined how these associations varied across coping tendencies. Avoidance and self-dysfunction were associated with a stronger statistical association between belongingness deficits and burdensomeness. In contrast, revenge motivation was associated with lower odds of reporting any suicidal ideation, a pattern interpreted as consistent with outward deflection/externalization, but likely at the cost of sustaining hostility. Conceptually, this embeds unforgiveness-based coping as moderators within an interpersonal framework. Clinically, the model suggests potential intervention targets: reducing avoidance, strengthening identity clarity, and pairing anger regulation with forgiveness-oriented work. Crucially, practitioners should recognize that revenge-driven externalization is an unstable deflection, not a cure. Methodologically, the study demonstrates the utility of Bayesian hurdle modeling and formal model comparison to test theoretical sequences in suicide research rigorously. At a systems level, these potential targets could be delivered through brief screening and low-intensity supports in campus and community services, using portable measures and hypotheses that enable cross-site replication and cross-cultural testing. Transparent analytic details further facilitate reproducibility and cumulative synthesis across settings.

## Data Availability

The data presented in this study are available on request from the corresponding author.
