# Supplementary material for: Mapping interpersonal-to-cognitive pathways with suicidal ideation: unforgiveness-based moderators within the interpersonal theory of suicide framework
Source: Front Psychol. 2026 Apr 9;17:1749224. doi: 10.3389/fpsyg.2026.1749224 (PMC13102621; doi:10.3389/fpsyg.2026.1749224)
Supplement: Supplementary file 1 [file Table_1.docx]

**Appendix A**

Appendix A.1. Prior Sensitivity Analysis and Prior Predictive Checks

This appendix provides supplementary results regarding the prior sensitivity analysis described in Section 3.4 of the main text. We compared the default weakly informative priors against a more regularizing ("narrow") prior set to assess the robustness of the model.

**Table A1.** Comparison of Prior Predictive Variance vs. Observed Data Variance

| Outcome Variable | Prior Specification | Prior Predictive Variance (Median) | Prior Predictive Variance (95% Range: Min–Max) | Observed Data Variance |
| --- | --- | --- | --- | --- |
| Perceived Burdensomeness | Default | 658.77 | 376.11 – 19509.38 | 1.00 |
|  | Narrow | 0.88 | 0.66 – 7.35 | 1.00 |
|  |  |  |  |  |
| Hopelessness | Default | 733.86 | 351.27 – 6511.63 | 1.00 |
|  | Narrow | 0.59 | 0.41 – 3.14 | 1.00 |
|  |  |  |  |  |
| Suicidal Ideation (Intensity) | Default | 765.97 | 348.70 – 9694.66 | 7.11 |
|  | Narrow | 2.49 | 1.87 – 5.84 | 7.11 |


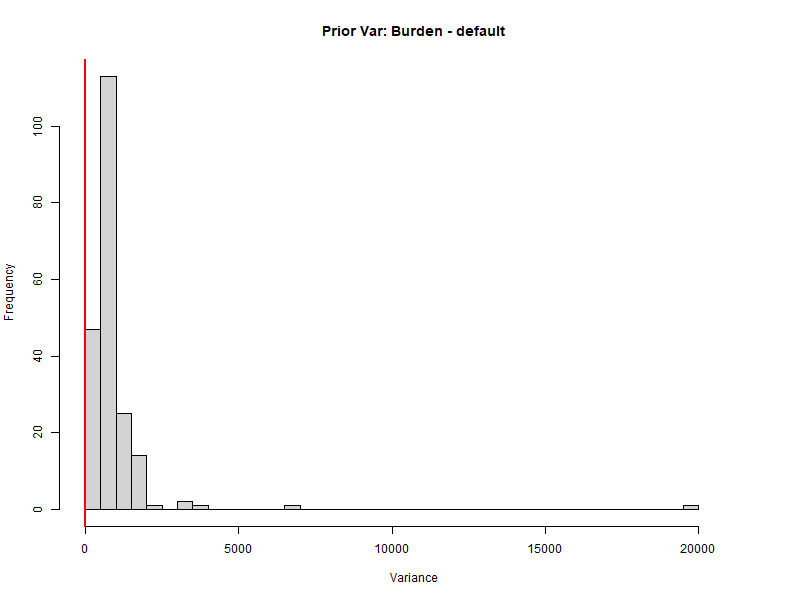


**Figure A1.** Prior Predictive Variance for Perceived Burdensomeness (Default Priors). The red vertical line represents the observed variance in the actual data. The default priors imply a very wide range of potential variance, allowing the data to drive the estimation.

**
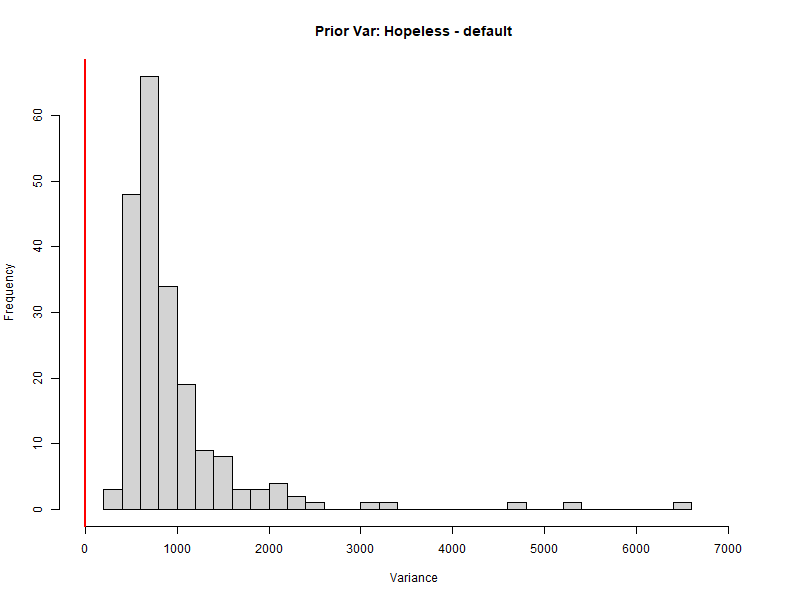

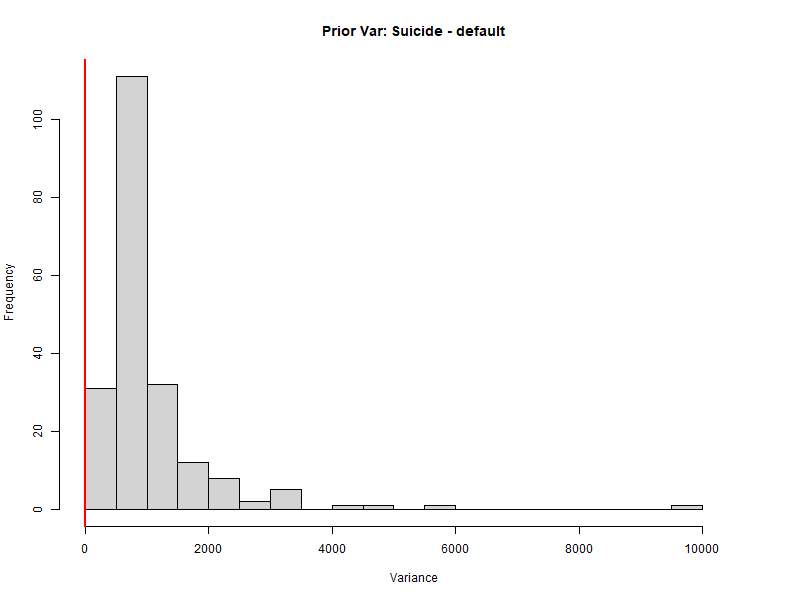
Figure A2.** Prior Predictive Variance for Hopelessness (Default Priors).

**Figure A3.** Prior Predictive Variance for Suicidal Ideation (Default Priors).


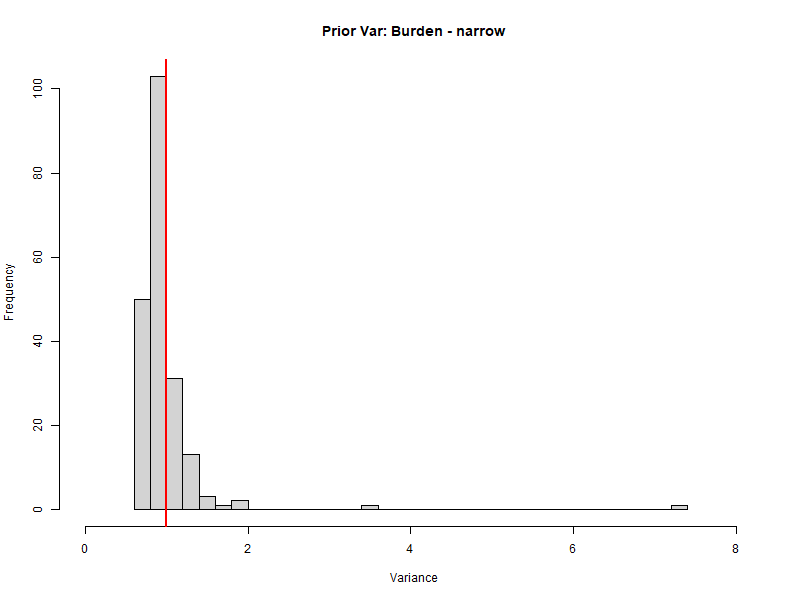
**
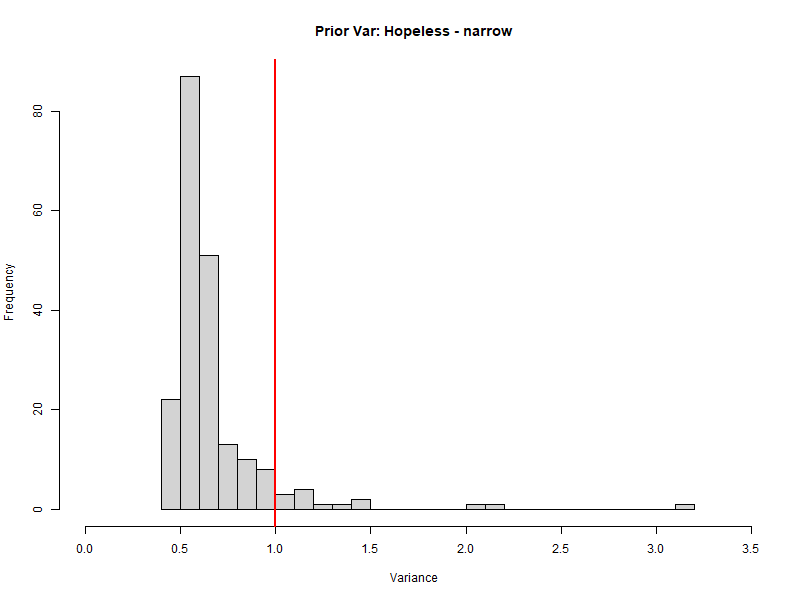
Figure A4.** Prior Predictive Variance for Perceived Burdensomeness (Narrow Priors). The narrow priors constrain the expected variance much closer to the standardized unit scale (1.0), testing model robustness.

**Figure A5.** Prior Predictive Variance for Hopelessness (Narrow Priors).

**
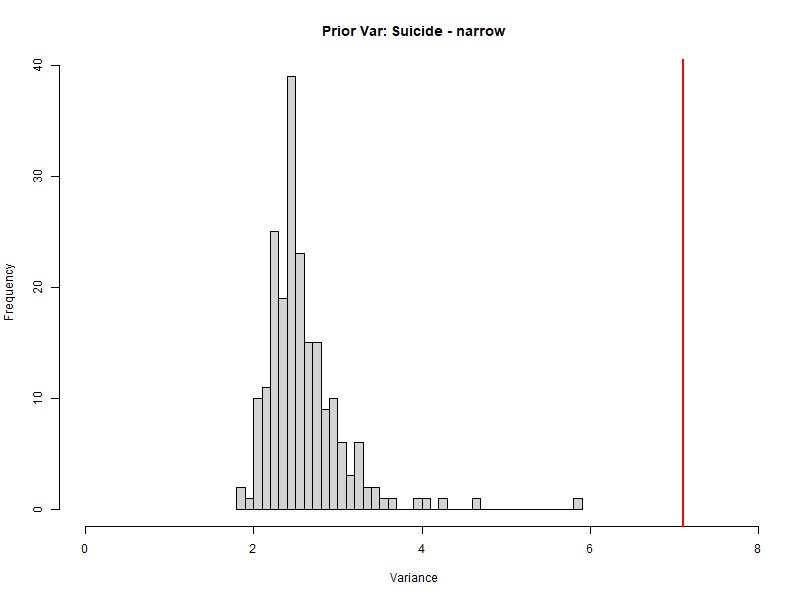
**

**Figure A6.** Prior Predictive Variance for Suicidal Ideation (Narrow Priors).

**Appendix B**

Appendix B.1. Full Model Results and Convergence Diagnostics

This appendix presents the complete parameter estimates for the adjusted Bayesian Hurdle Log-normal model. Unlike the summarized results in the main text, this table includes estimates for all levels of categorical covariates (e.g., specific religious affiliations, parental marital status categories) and convergence diagnostics (R-hat, Bulk ESS, Tail ESS) for every parameter to demonstrate model stability.

**Table B1.** Full Parameter Estimates, 95% Credible Intervals, and Convergence Diagnostics for the Adjusted Bayesian Hurdle Lognormal Model

| Parameter | Estimate [95% CrI] | R-hat | Bulk ESS | Tail ESS |
| --- | --- | --- | --- | --- |
|  | Odds Ratio (OR) |  |  |  |
| Part 1: Hurdle Component (Probability of Any Ideation) |  | |  |  |
| Main Predictors | |  |  |  |
| Thwarted Belongingness (TB) | 1.32 [0.77, 2.31] | 1 | 15347 | 9775 |
| Perceived Burdensomeness (PB) | 1.93 [1.07, 3.71] | 1 | 15432 | 9657 |
| Hopelessness (HL) | 1.90 [.65, 5.83] | 1 | 15458 | 8126 |
| Revenge Motivation (RM) | 1.01 [.69, 1.45] | 1 | 12487 | 10029 |
| Avoidance Motivation (AM) | 1.04 [.64, 1.68] | 1 | 14780 | 9797 |
| Self-Dysfunction (SF) | 2.36 [.75, 7.82] | 1 | 15648 | 8877 |
| **Interactions** | |  |  |  |
| TB × Revenge Motivation | **.51 [.34, 0.72]** | 1 | 11217 | 8620 |
| TB × Avoidance Motivation | 1.27 [.78, 2.01] | 1 | 12440 | 9915 |
| TB × Self-Dysfunction | 1.40 [.61, 3.46] | 1 | 14954 | 9997 |
| **Covariates** | |  |  |  |
| Age | .98 [.86, 1.10] | 1 | 17644 | 9386 |
| Gender (Male vs. Female) | 2.68 [1.05, 7.26] | 1 | 18143 | 9035 |
| Family Income (vs. Level 5/Ref) ᵃ | | | |  |
| Level 1 | .00 [.00, 3.21] | 1 | 3381 | 2813 |
| Level 2 | .00 [.00, 4.73] | 1 | 3341 | 2839 |
| Level 3 | .00 [.00, 2.13] | 1 | 3347 | 2835 |
| Level 4 | .00 [.00, 1.39] | 1 | 3347 | 2821 |
| Education (vs. Primary/Ref) | | |  |  |
| Secondary | .00 [.00, 2.90] | 1 | 4576 | 3669 |
| College | .00 [.00, 0.29] | 1 | 4753 | 3584 |
| Undergraduate | .00 [.00, 0.48] | 1 | 4805 | 3667 |
| Religion (vs. None/Ref) | | |  |  |
| Catholic | 3.40 [0.19, 139.75] | 1 | 18194 | 8121 |
| Christian | 1.25 [0.41, 4.17] | 1 | 19111 | 9466 |
| Buddhism | 22.03 [1.30, 992.78] | 1 | 16920 | 7896 |
| Taoism ᵇ | > 1000 [Wide CI] | 1 | 6355 | 5264 |
| Other | .02 [.00, 1.09] | 1 | 13936 | 9207 |
| Parental Marital Status (vs. Married) | | | |  |
| Remarriage | .89 [.06, 31.25] | 1 | 16795 | 8187 |
| Divorce | 2.39 [.64, 10.77] | 1 | 18705 | 9184 |
| Separation | .08 [.00, 1.25] | 1 | 16430 | 8094 |
| Cohabitation ᵇ | > 1000 [Wide CI] | 1 | 7261 | 6463 |
| Other | .67 [0.11, 4.90] | 1 | 18061 | 9154 |
|  |  |  |  |  |
|  |  |  |  |  |
| Part 2: Intensity Component (Severity of Ideation) | Geom. Mean Ratio (GMR) | | |  |
| Main Predictors | |  |  |  |
| Thwarted Belongingness (TB) | 1.01 [.89, 1.15] | 1 | 14758 | 9701 |
| Perceived Burdensomeness (PB) | 1.19 [1.07, 1.33] | 1 | 16077 | 9569 |
| Hopelessness (HL) | 1.19 [.92, 1.53] | 1 | 14315 | 9564 |
| Revenge Motivation (RM) | .98 [.90, 1.07] | 1 | 15704 | 9359 |
| Avoidance Motivation (AM) | 1.10 [.96, 1.26] | 1 | 16478 | 9803 |
| Self-Dysfunction (SF) | 1.18 [.91, 1.52] | 1 | 15668 | 9324 |
| **Interactions** | |  |  |  |
| TB × Revenge Motivation | 1.03 [.95, 1.12] | 1 | 14547 | 10388 |
| TB × Avoidance Motivation | .93 [.85, 1.02] | 1 | 14336 | 9651 |
| TB × Self-Dysfunction | .96 [.80, 1.14] | 1 | 18672 | 8961 |
| **Covariates** | |  |  |  |
| Age | .97 [.94, 1.00] | 1 | 16189 | 9553 |
| Gender (Male vs. Female) | .85 [.67, 1.10] | 1 | 16126 | 9822 |
| Family Income (vs. Level 5/Ref) ᵃ | | | |  |
| Level 1 | .77 [.45, 1.34] | 1 | 6503 | 7579 |
| Level 2 | .90 [.53, 1.55] | 1 | 6471 | 6805 |
| Level 3 | 1.09 [.63, 1.89] | 1 | 6258 | 8124 |
| Level 4 | 1.06 [.62, 1.81] | 1 | 6492 | 7628 |
| Education (vs. Primary/Ref) | | |  |  |
| Secondary | 1.07 [.60, 1.90] | 1 | 10369 | 9736 |
| College | 1.32 [.86, 2.02] | 1 | 10111 | 9695 |
| Undergraduate | 1.27 [.70, 2.28] | 1 | 10442 | 9217 |
| Religion (vs. None/Ref) | | |  |  |
| Catholic | 1.18 [.65, 2.16] | 1 | 18656 | 9257 |
| Christian | 1.12 [.84, 1.49] | 1 | 15013 | 9462 |
| Buddhism | 1.06 [.63, 1.76] | 1 | 17057 | 9762 |
| Taoism | .85 [.46, 1.58] | 1 | 17073 | 9301 |
| Other | 1.81 [.57, 5.63] | 1 | 16818 | 9419 |
| Parental Marital Status (vs. Married) | | | |  |
| Remarriage | 1.18 [.64, 2.22] | 1 | 17607 | 9090 |
| Divorce | 1.27 [.95, 1.71] | 1 | 17514 | 8803 |
| Separation | 1.36 [.49, 3.91] | 1 | 19387 | 9392 |
| Cohabitation | .63 [.37, 1.07] | 1 | 16241 | 8989 |
| Other | .87 [.57, 1.31] | 1 | 18442 | 9383 |
| ***Note***. OR = Odds Ratio; GMR = Geometric Mean Ratio; CrI = Credible Interval; ESS = Effective Sample Size.  ᵃ Family Income: In this comprehensive model validation, Family Income was treated as a categorical variable to inspect potential non-linear effects across income levels, differing from the simplified continuous specification used in the main text.ᵇ Wide Intervals: Estimates for these specific subgroups in the Hurdle component exhibited extremely wide credible intervals due to quasi-complete separation (low or zero counts of the outcome event in these small subgroups), a known phenomenon in logistic regression components. However, the model remained convergent (R-hat = 1.00). | | | | |

**Appendix C**

Appendix C1: Analytical Software and Estimation Specifications

**Table C1.** Bayesian Estimation Settings

| **Setting** | **SEM (blavaan)** | **Hurdle lognormal (brms)** |
| --- | --- | --- |
| Computational backend | Stan (via rstan) | Stan (via brms) |
| Number of chains | 2 | 4 |
| Warmup iterations per chain | 1,000 | 1,000 |
| Post-warmup samples per chain | 5,000 | 3,000 |
| Total posterior draws | 10,000 | 12,000 |
| Seed | 1234 | 1234 |
| adapt_delta | .99 | .99 |
| max_treedepth | 15 | 15 |
| Convergence: R̂ threshold | ≤ 1.01 | ≤ 1.01 |
| Convergence: minimum ESS | > 400 | > 400 |

**Table C2. Prior Specifications**

| **Parameter** | **Default (primary)** | **Narrow (sensitivity)** |
| --- | --- | --- |
| **Hurdle lognormal (brms)** |  |  |
| Regression slopes (β) | Normal(0, 1.0) | Normal(0, 0.2) |
| Intercepts (mu) | Student-t(3, 0, 2.5) | Student-t(3, 0, 1.0) |
| Hurdle intercept (hu) | Normal(0, 1.0) | Normal(0, 1.0) |
| **Bayesian SEM (blavaan)** |  |  |
| Path coefficients | blavaan Stan defaults | Normal(0, 0.2) |
| Intercepts | blavaan Stan defaults | Normal(0, 0.5) |

**Table C3. Key R Packages and Versions**

| **Package** | **Purpose** | **Version** |
| --- | --- | --- |
| R | Statistical computing environment | 4.5.1 |
| blavaan | Bayesian SEM (Stan backend) | 0.5-9 |
| brms | Bayesian hurdle lognormal regression | 2.23.0 |
| lavaan | SEM engine (used by blavaan) | 0.6-21 |
| loo | WAIC, LOO-IC, Pareto-k diagnostics | 2.8.0 |
| bayestestR | Probability of direction, ROPE | 0.17.0 |
| posterior | Posterior draw extraction and summaries | 1.6.1 |
| rstan | Stan computational backend | 2.32.7 |
| bridgesampling | Marginal likelihood / Bayes factors | 1.1-2 |
| EValue | Sensitivity to unmeasured confounding | 4.1.4 |
| psych | Cronbach's α, descriptive statistics | 2.5.6 |
| dplyr | Data wrangling | 1.1.4 |
| ggplot2 | Visualization | 4.0.1 |
| haven | SPSS data import | 2.5.5 |
| *Note.* All analyses were run under R 4.5.1 on Windows 11 x64 (platform: x86_64-w64-mingw32). The full analysis script is available from the corresponding author upon request. | | |
